# ﻿Rhagophthalmidae Olivier, 1907 (Coleoptera, Elateroidea): described genera and species, current problems, and prospects for the bioluminescent and paedomorphic beetle lineage

**DOI:** 10.3897/zookeys.1126.90233

**Published:** 2022-11-01

**Authors:** Robin Kundrata, Johana Hoffmannova, Kevin R. Hinson, Oliver Keller, Gabriela Packova

**Affiliations:** 1 Department of Zoology, Faculty of Science, Palacky University, 17. listopadu 50, 77900, Olomouc, Czech Republic Palacky University Olomouc Czech Republic; 2 EpiLogic GmbH Agrarbiologische Forschung und Beratung, Hohenbachernstr. 19–21, 85354, Freising, Germany EpiLogic GmbH Agrarbiologische Forschung und Beratung Freising Germany; 3 Florida State Collection of Arthropods, Florida Department of Agriculture and Consumer Services, P.O. Box 147100, Gainesville, FL, 32614-7100, USA Florida Department of Agriculture and Consumer Services Gainesville United States of America

**Keywords:** Catalogue, classification, Drilidae, Lampyridae, neoteny, Oriental Region, Phengodidae

## Abstract

Rhagophthalmidae are a small beetle family known from the eastern Palaearctic and Oriental realms. Rhagophthalmidae are closely related to railroad worms (Phengodidae) and fireflies (Lampyridae) with which they share highly modified paedomorphic females and the ability to emit light. Currently, Rhagophthalmidae include 66 species classified in the following 12 genera: *Bicladodrilus* Pic, 1921 (two spp.), *Bicladum* Pic, 1921 (two spp.), *Dioptoma* Pascoe, 1860 (two spp.), *Diplocladon* Gorham, 1883 (two spp.), *Dodecatoma* Westwood, 1849 (eight spp.), *Falsophrixothrix* Pic, 1937 (six spp.), *Haplocladon* Gorham, 1883 (two spp.), *Menghuoius* Kawashima, 2000 (three spp.), *Mimoochotyra* Pic, 1937 (one sp.), *Monodrilus* Pic, 1921 (two spp. in two subgenera), *Pseudothilmanus* Pic, 1918 (two spp.), and *Rhagophthalmus* Motschulsky, 1854 (34 spp.). The replacement name *Haplocladongorhami* Kundrata, **nom. nov.** is proposed for *Diplocladonhasseltii* Gorham, 1883b (described in subgenus Haplocladon) which is preoccupied by *Diplocladonhasseltii* Gorham, 1883a. The genus *Reductodrilus* Pic, 1943 is tentatively placed in Lampyridae: Ototretinae. Lectotypes are designated for *Pseudothilmanusalatus* Pic, 1918 and *P.marginalis* Pic, 1918. Interestingly, in the eastern part of their distribution, Rhagophthalmidae have remained within the boundaries of the Sunda Shelf and the Philippines demarcated by the Wallace Line, which separates the Oriental and Australasian realms. This study is intended to be a first step towards a comprehensive revision of the group on both genus and species levels. Additionally, critical problems and prospects for rhagophthalmid research are briefly discussed.

## ﻿Introduction

**Figure 1.**
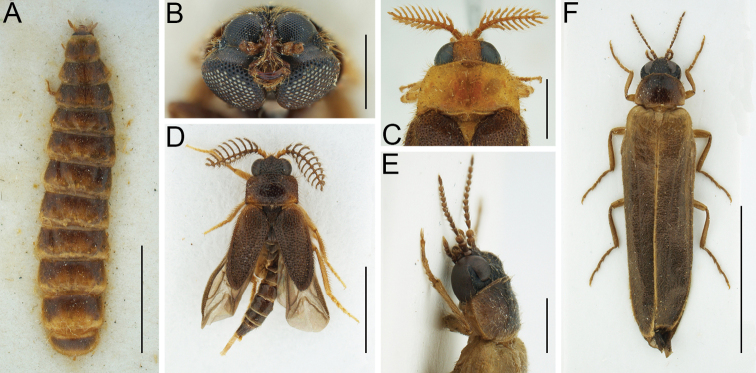
Morphology of Rhagophthalmidae**A** habitus of *Dioptomaadamsii* from Sri Lanka (syntype of *D.greeni*), female, NHMUK, dorsal view **B** head of *Dioptomaadamsii* from Sri Lanka, male, NHMUK, frontal view **C** head and pronotum of *Diplocladonhasseltiihasseltii* from Indonesia, male, SDEI, dorsal view **D** habitus of *Falsophrixothrix* sp. from Indonesia, male, NHMUK, dorsal view **E** head and pronotum of *Rhagophthalmus* sp. from China, male, first author’s collection, lateral view **F** habitus of *Rhagophthalmus* sp. from China, male, first author’s collection, dorsal view. Scale bars: 5.0 mm (**A, F**); 1.0 mm (**B, E**); 2.0 mm (**C, D**).

Rhagophthalmidae are a small elateroid family distributed in South, East, and Southeast Asia (Wittmer 1979; Kawashima et al. 2010; Kundrata and Bocak 2011a; Kazantsev 2012). Soft-bodied males are capable of flight, whereas all known females are strongly paedomorphic and remain larva-like as adults (Fig. 1). Predaceous larvae occur in soil and leaf litter where they feed on millipedes. Both larvae and adults are bioluminescent, although the biology and ecology of most species are unknown (Li and Liang 2008; Kawashima et al. 2010). Rhagophthalmidae have a convoluted history of classification. Most genera were originally placed either in Lampyridae or the widely defined Drilidae (currently Drilini in Elateridae: Agrypninae; Kundrata and Bocak 2011b). The separate family Rhagophthalmidae was proposed by Olivier (1907, 1910) for genera which had antennae with 12 antennomeres and more or less emarginate eyes. However, since their erection, the composition and classification of Rhagophthalmidae have varied greatly, and various authors have recognized 3–11 genera in the group. At various times, the majority of Rhagophthalmidae have been considered either a subgroup of Lampyridae (e.g., McDermott 1964, 1966) or Phengodidae (Crowson 1972; Lawrence and Newton 1995; Bocak 2007), or a separate family close to one of the two above-mentioned families (Olivier 1910; Winkler 1925; Wittmer and Ohba 1994). Recent phylogenomic approaches suggest Rhagophthalmidae are sister to Phengodidae, and both are closely related to Lampyridae, Sinopyrophoridae, and Elateridae (Zhang et al. 2018; Douglas et al. 2021; Kusy et al. 2021; Cai et al. 2022).

The early history of Rhagophthalmidae systematic research dates back to 1849, when Westwood (1849) described *Dodecatoma* Westwood, 1849 based on a single species from India. Motschulsky (1854) then described *Rhagophthalmus* Motschulsky, 1854 based on one species from China, and Pascoe (1860, 1862) added *Dioptoma* Pascoe, 1860 and *Ochotyra* Pascoe, 1862 from Bangladesh and India, respectively. While *Dodecatoma* was placed in the widely delimited Drilidae, the remaining genera were classified in Lampyridae (e.g., Gemminger 1869). Gorham (1883a, b) described Diplocladon Gorham, 1883 and its subgenus Haplocladon Gorham, 1883, both from Indonesia, and placed them in Drilidae.

Several new species of *Rhagophthalmus* from Southeast Asia, India, and China were added by Olivier (1885) and Fairmaire (1889, 1896, 1899). Gorham (1895) described the second species of *Dodecatoma* from India, and classified *Dioptoma*, *Diplocladon*, *Dodecatoma*, *Haplocladon* (originally as a subgenus), and *Ochotyra* in Drilinae. He later described a second species of *Haplocladon*, which was collected in India (Gorham 1903). Olivier (1907) erected the family Rhagophthalmidae for *Dioptoma*, *Ochotyra*, and *Rhagophthalmus*. In 1910, he provided the first catalogues for Rhagophthalmidae and Drilidae (Olivier 1910), with the latter including *Diplocladon* (with *Haplocladon* as a synonym) and *Dodecatoma*. Jakobson (1911) included many soft-bodied groups, including “Rhagophthalmini”, in his “Cantharididae”. Olivier (1912) revised *Rhagophthalmus* and recognized 12 species, five of which were newly described from China and Sri Lanka. Gahan in Morice (1913) reported a new species of *Dioptoma* from Sri Lanka.

Many new taxa currently belonging to Rhagophthalmidae were then described by the French coleopterist Maurice Pic, a person famous for his usually short and uninformative descriptions (e.g., Villiers 1958; Bezděk and Regalin 2015). Pic described the following taxa from Asia: one new species of *Dioptoma* and four species of *Rhagophthalmus* from India, Sri Lanka, China, and Indochina (Pic 1916, 1917, 1925a, b); genus Pseudothilmanus Pic, 1918, with its monotypic subgenus Drilothilmanus Pic, 1918 from northern India (Pic 1918); genus *Bicladodrilus* Pic, 1921, with two species from the Philippines and Vietnam (Pic 1921a, 1923); genus *Bicladum* Pic, 1921, with two species from Borneo and Sumatra (Pic 1921b, 1930a); a new variety and a new species of *Dodecatoma* from Indonesia and the Philippines, respectively (Pic 1921b, 1924); a new variety of *Diplocladon* from Indonesia (Pic 1921b); a new genus *Monodrilus* Pic, 1921 from Indonesia (Pic 1921b) and subsequently the monotypic subgenus Dodecatomorpha Pic, 1928 from Vietnam (Pic 1928); and a monotypic *Mimoochotyra* Pic, 1937 from Indonesia. Pic (1937) also erected *Falsophrixothrix* Pic, 1937 for two species from Indonesia, one of which was already described by Pic in the genus *Phrixothrix* Olivier, 1909 (currently in Phengodidae; Pic 1914).

Later, Wittmer (1939, 1944) added another three species from Indonesia and Singapore to *Falsophrixothrix*, with one being new and two transferred from *Phrixothrix* (Olivier 1911; Pic 1921a). Wittmer (1944) published a comprehensive catalogue of genera and species in Drilidae in which he listed many genera that are currently in Rhagophthalmidae, i.e., *Bicladodrilus*, *Bicladum* (as *Bicladon* [sic!]), *Diplocladon* (with *Haplocladon* as a synonym), *Dodecatoma*, *Falsophrixothrix*, *Mimoochotyra*, *Monodrilus*, and *Pseudothilmanus*. Pic (1951) described an additional species of *Falsophrixothrix* from Vietnam. In his major works on Lampyridae, McDermott (1964, 1966) included *Dioptoma*, *Mimoochotyra* (as *Mimochotyra* [sic!]), *Ochotyra*, and *Rhagophthalmus* in the subfamily Rhagophthalminae.

Crowson (1972) redefined Drilidae to include only a few core genera. Although Crowson excluded the majority of genera from Drilidae, he did not suggest any family placement for many, which left them in an uncertain position. Crowson (1972) also redefined Phengodidae by including *Cydistus* Bourgeois, 1885 as well as genera which are currently in Rhagophthalmidae, i.e., *Dioptoma*, *Diplocladon*, *Falsophrixothrix*, and *Rhagophthalmus*. Lawrence and Newton (1995) distinguished the subfamily Rhagophthalminae within Phengodidae, and included the genera *Cydistus*, *Dioptoma*, *Diplocladon*, *Dodecatoma*, *Falsophrixothrix*, *Mimoochotyra* (as *Mimochotrya* [sic!]), *Ochotyra* (as *Ochotrya* [sic!]), and *Rhagophthalmus*. Other major works on Rhagophthalmidae were those by Walter Wittmer, who described three new species of *Dodecatoma* from Afghanistan, India, and Nepal (Wittmer 1979, 1995), synonymized *Ochotyra* with *Rhagophthalmus* (Wittmer and Ohba 1994), and described eight new species of *Rhagophthalmus* from China, Japan, and Myanmar (Wittmer and Ohba 1994; Wittmer 1997).

Kawashima (1998) described the morphology of a larviform adult female of *Rhagophthalmus*. He also erected *Menghuoius* Kawashima, 2000 for two Chinese species originally classified in *Rhagophthalmus*, and later described the third species of that genus from Myanmar (Kawashima 2000, 2002). Kawashima and Satô (2001) described three species of *Rhagophthalmus* from Myanmar, Taiwan, and Thailand, and Kawashima and Sugaya (2003) added an additional new species from Taiwan. Branham and Wenzel (2003) studied the evolution of bioluminescence in the soft-bodied elateroids (i.e., “cantharoids”) and confirmed that *Rhagophthalmus* is closely related to *Dioptoma* and *Diplocladon*. Li and Liang (2008) described the morphology of a larviform adult female of *Diplocladon* from China. Li et al. (2008a) described two new species of *Rhagophthalmus* from China, provided information on the morphology and distribution for several other species, and provided a distribution map for all species in China and surrounding regions.

In the Rhagophthalmidae chapter of the Handbook of Zoology, Kawashima et al. (2010) included only *Dioptoma*, *Diplocladon*, *Dodecatoma*, *Menghuoius*, *Mimoochotyra* (as *Mimochotyra* [sic!]), and *Rhagophthalmus*. Kundrata and Bocak (2011a) revised the long-neglected genus *Pseudothilmanus* (with its subgenus Drilothilmanus, which they synonymized with *Pseudothilmanus*), added it to Rhagophthalmidae, and also listed *Bicladodrilus*, *Bicladum* (as *Bicladon* [sic!]), *Dioptoma*, *Diplocladon*, *Dodecatoma*, *Falsophrixothrix*, *Mimoochotyra* (as *Mimochotyra* [sic!]), *Monodrilus*, *Reductodrilus*, and *Rhagophthalmus* (including *Menghuoius* and *Ochotyra*). Ho et al. (2012) described two new species of *Rhagophthalmus* from Taiwan. Kazantsev (2012) described two species of *Dodecatoma* from India and Nepal. Most recently, Yiu (2017) described a new species of *Diplocladon* and a new species of *Rhagophthalmus* from Hong Kong. Roza (2020) added information on the morphology and distribution of *Pseudothilmanus*.

Besides research on the diversity, systematics, and morphology of Rhagophthalmidae, many studies in the 21^st^ century have focused on their bioluminescence (Ohmiya et al. 2000; Ohba 2004a; Chen et al. 2010; Oba et al. 2011; Oba 2015; Liu et al. 2020) and embryogenesis (Kobayashi et al. 2001, 2002, 2003). Additionally, the rapid development of molecular phylogenetic methods in the last decades has enabled scientists to test the phylogenetic placement of Rhagophthalmidae within Elateroidea using one or several markers (e.g., Suzuki 1997; Bocakova et al. 2007; Sagegami-Oba et al. 2007; Stanger-Hall et al. 2007; Kundrata and Bocak 2011b; Kundrata et al. 2014; McKenna et al. 2015), mitogenomes (Li et al. 2007; Amaral et al. 2016; Chen et al. 2019), or a phylogenomic approach (Zhang et al. 2018; Amaral et al. 2019; Douglas et al. 2021; Kusy et al. 2021; Cai et al. 2022).

Despite the long history of rhagophthalmid systematic research, we lack a comprehensive study which would summarize all relevant information of all genera and species in the group. Therefore, in this study, we provide an annotated catalogue of genera and species of Rhagophthalmidae, including information on their synonyms, type material, distribution, and bibliography. We believe this study will serve as a robust framework for subsequent taxonomic revisions of all genera in addition to studies devoted to diversity, evolution, nature conservation, and ecology of the group.

## ﻿Materials and methods

Names of family-, genus-, and species-group taxa are given with the name of the author, and the year and page of publication. The page given is the page where the taxon name and description are printed. The year and page given for the incorrect subsequent spellings are the first year and page in which they are used. Incorrect subsequent spellings not in prevailing usage are unavailable (ICZN 1999, Art. 33.3). Complete data and comments for genus-group names are presented with the lowest-rank name, i.e., subgenus rather than genus, since these criteria follow the Principle of Coordination (ICZN 1999, Art. 36.1 and 43.1).

We provide the type species for each genus-group name, including information on its designation. We follow Recommendation 73F of the Code (ICZN 1999) and provide lectotype designations to fix the species identity for two species of *Pseudothilmanus* Pic, 1918. These species were originally described based on an unknown number of specimens, then redescribed (Kundrata and Bocak 2011a) under the assumption that the original descriptions were based only on holotypes. We do not provide lectotype designations for species in other genera, as they must first be revised in detail. Under each name, the currently valid name is listed first, followed by synonyms in chronological order.

Misspellings and unavailable names are followed by a colon “:”. We list all relevant references known to us for all genera, as well as for the family Rhagophthalmidae, particularly those that include information on systematics, classification, phylogeny, biology, and ecology. Since PhD or any other student theses are not officially published in the sense of the Code (ICZN 1999), we list only the relevant works (i.e., Ho 2002; Jeng 2008; Roza 2022) at the end of the Literature sections under each genus and species. Dates of publications and exact bibliographic references (especially problematic ones, often not cited uniformly by researchers) are taken from the following comprehensive general works: Chandler (2000); Bouchard et al. (2011); Bousquet (2016); and Evenhuis (2020). For the date of publication of F. P. Pascoe’s description of the genus *Dioptoma* (Pascoe 1860), we follow Evenhuis (2020).

### ﻿Type depositories

**ESRI**Endemic Species Research Institute, JiJi, Nantou, Taiwan, Taiwan

**ICM** Insect Center, Moscow, Russia

**KNHMZ**Kunming Natural History Museum of Zoology, Kunming Institute of Zoology, Chinese Academy of Sciences, Kunming, China

**MNHN**Museum National d’Histoire Naturelle, Paris, France

**MSNG**Museo Civico di Storia Naturale, Genova, Italy

**MZB** Bogor Zoology Museum, Bogor, Indonesia

**NHMB**Naturhistorisches Museum, Basel, Switzerland

**NHMUK**Natural History Museum, London, The United Kingdom

**NKME** Naturkundemuseum Erfurt, Germany

**NMNS**Department of Entomology, National Taiwan University, Taiwan

**NTU** Department of Entomology, National Taiwan University, Taipei, Taiwan

**NWU** Nagoya Women’s University, Nagoya, Japan

**PCIK** collection of I. Kawashima, Yokosuka-shi, Kanagawa, Japan

**RMNH**Naturalis Biodiversity Center, Leiden, The Netherlands

**SMNH**Swedish Museum of Natural History, Stockholm, Sweden

**SMNS**Staatliches Museum für Naturkunde, Stuttgart, Germany

**TARI**Taiwan Agricultural Research Institute, Taichung, Taiwan

**TLES** Insect Museum, Tai Lung Experimental Station, Hong Kong, China

**YCM**Yokosuka City Museum, Yokosuka, Japan

**ZMM** Zoological Museum of M.V. Lomonosov State University, Moscow, Russia

## ﻿Systematics

### ﻿ Rhagophthalmidae

Taxon classificationAnimaliaColeopteraRhagophthalmidae

Olivier, 1907

D27F3E3C-1F04-5C21-94A8-DF877076DE15

Rhagophthalmidae E. Olivier, 1907: 63. Type genus. Rhagophthalmus Motschulsky, 1854.
Rhagophtalmidae : Junk 1912: 24 [unavailable name, incorrect subsequent spelling not in prevailing usage].
Rhagophthalmidae : Blair in Gahan 1925: vi [unavailable name, incorrect subsequent spelling not in prevailing usage].
Rhagopthalmidae: Harvey 1952: 389 [unavailable name, incorrect subsequent spelling not in prevailing usage].
Rhagophthalidae: Ohba 1998: 2 [unavailable name, incorrect subsequent spelling not in prevailing usage].
Rhagophthammidae: Suzuki and Kobayashi 2009: 31 [unavailable name, incorrect subsequent spelling not in prevailing usage].

#### Literature.

Olivier (1907: 1, 63): catalogue; Lefroy (1909: 327): catalogue; Olivier (1910: 3): catalogue; Jakobson (1911: 662, 687): catalogue [as Rhagophthalmini]; Junk (1912: 24): bibliography [as Rhagophtalmidae [sic!]]; Olivier (1912: 467): revision of *Rhagophthalmus*; Blair (1915a: 411): bioluminescence; Pic (1923: 25): catalogue; Gahan (1925: vi): remark [as Phagophthalmidae [sic!]; attributed to KG Blair]; Handlirsch (1925: 589): catalogue [as Rhagophthalmini]; Winkler (1925: 522): catalogue; Ridley (1934: 58): larval biology and morphology; Pic (1937: 137): genus description; Harvey (1952: 389, 450): remark, bioluminescence [also as Rhagopthalmidae [sic!], also as Rhagopthalminae [sic!]]; Brues et al. (1954: 565): classification; Crowson (1955: 68): remark, morphology [as Rhagophthalminae]; Harvey (1955: 19): checklist, bioluminescence; Raj (1957: 788): larval biology; McDermott (1964: 49): revision [as Rhagophthalminae]; McDermott (1966: preface (unnumbered),121): catalogue, distribution [as Rhagophthalminae]; Mikšić and Mikšić (1966: 31): remark [as Rhagophthalminae]; Nakane (1968: 3): remark [as Rhagophthalminae]; Crowson (1972: 50): classification, morphology [as Rhagophthalminae]; McElroy et al. (1974: 415): remark [as Rhagopthalmidae [sic!]]; Lawrence (1982: 512): remark; Haneda (1985: 167): bioluminescence [as Rhagopthalmidae [sic!]]; Herring (1987: 158): checklist [as Rhagophthalminae]; LeSage (1991: 424): remark [also as Rhagophthalminae]; Wittmer and Ohba (1994: 341): taxonomy, biology; Lawrence and Newton (1995: 857): catalogue, review [as Rhagophthalminae]; Chen and Ho (1996: 46): distribution; Ohba et al. (1996a: 1): morphology, biology; Ohba (1997a: 5): checklist; Ohba (1997c: 51): breeding; Suzuki (1997: 11, 38): phylogeny, biology [also as Rhagophthalminae]; Wittmer (1997: 257): species descriptions; Chen and Ho (1998: 34): bioluminescence; Ohba (1998: 2): biology [also as Rhagophthalidae [sic!]]; Costa et al. (1999: 22): remark [as Rhagophthalminae]; Goto and Kawashima (2000: 141): distribution; Jeng et al. (2000: 316): remark; Kawashima (2000: 131): genus description; Kim et al. (2000: 214): molecular phylogeny; Ohmiya et al. (2000: 32): luciferase; Branham and Wenzel (2001: 565): phylogeny [also as Rhagophthalminae and Rhagopthalmidae [sic!]]; Kawashima and Satô (2001: 423): species descriptions; Kobayashi et al. (2001: 1): embryogenesis, morphology [also as Rhagophthalminae]; Hua (2002: 71): catalogue; Kawashima (2002: 487): species description; Kobayashi et al. (2002: 1): embryogenesis, morphology [also as Rhagophthalminae]; Branham and Wenzel (2003: 3): phylogeny; Chen (2003: 52): morphology, bioluminescence; Hayashi and Suzuki (2003: 4): biology, morphology, phylogeny, figure of mating; Kawashima and Sugaya (2003: 353): species description; Kawashima et al. (2003: 255): catalogue; Kobayashi et al. (2003: 19): embryogenesis, morphology; DeCock (2004: 341): bioluminescence; Ohba (2004a: 225): bioluminescence, biology; Lau and Meyer-Rochow (2006: 19): eye morphology; Li et al. (2006: 817): molecular phylogeny; Arnoldi et al. (2007: 2): molecular phylogeny, remark; Bocak (2007: 224): catalogue [as Rhagophthalminae]; Bocakova et al. (2007: 477): molecular phylogeny [also as Rhagophthalminae]; Hunt et al. (2007: 1915): molecular phylogeny; Li et al. (2007: 197): mitochondrial genome, phylogeny [also as Rhagophthalminae]; Sagegami-Oba et al. (2007: 110): molecular phylogeny [also as Rhagophthalminae]; Stanger-Hall et al. (2007: 38): molecular phylogeny; Bocak et al. (2008: 2021): molecular phylogeny; Li and Liang (2008: 109): female morphology; Li et al. (2008a: 259): species descriptions, distribution [also as Rhagophthalminae]; Li et al. (2008b: 494): review [also as Rhagophthalminae]; Bogahawatta et al. (2009: 5): distributional remark [as Rhagophthalminae]; Levkanicova and Bocak (2009: 212): molecular phylogeny; Suzuki and Kobayashi (2009: 30): embryogenesis [also as Rhagophthalminae and Rhagophthammidae [sic!]]; Chen et al. (2010: 196): biology, bioluminescence; Kawashima et al. (2010: 135): book chapter [also as Rhagophthalminae]; Lawrence et al. (2010a: 5): classification; Lawrence et al. (2010b: 165): remark; Bouchard et al. (2011: 326): family-group names catalogue; Kundrata and Bocak (2011a: 57): revision of *Pseudothilmanus*; Kundrata and Bocak (2011b: 364): molecular phylogeny [also as Rhagophthalminae]; Lawrence et al. (2011: 7): phylogeny; Oba et al. (2011: 775): biology, bioluminescence [also as Rhagophthalminae]; Yiu (2011a: 14): remark; Yiu (2011b: 20): bioluminescence, larva; Amaral et al. (2012: 1262): luciferase, phylogeny [as Rhagophthalminae]; Ho et al. (2012: 1): species descriptions; Johnson et al. (2012: 178): ICZN case; Kazantsev (2012: 349): species descriptions; Timmermans and Vogler (2012: 299): remark, molecular phylogeny; Kundrata et al. (2013: 201): molecular phylogeny; Yiu (2013: 101): remark, bioluminescence; Amaral et al. (2014: 415): molecular phylogeny; Bocak et al. (2014: 103): molecular phylogeny; Hosoe et al. (2014: 331): biology; ICZN (2014: 195): ICZN case; Kundrata et al. (2014: 163): molecular phylogeny; Li et al. (2015: 269): catalogue; Martin et al. (2015: 516): molecular phylogeny; McKenna et al. (2015: 843): molecular phylogeny [also as Rhagopthalmidae [sic!]]; Oba (2015: 99): bioluminescence; Amaral et al. (2016: 255): molecular phylogeny; Bocak et al. (2016: 2): molecular phylogeny; Kundrata et al. (2016: 293): molecular phylogeny; Lawrence (2016: 17): classification; Wijekoon et al. (2016: 69): checklist [also as Rhagophthalminae]; Amaral et al. (2017a: 674): mitogenome, phylogeny; Kundrata et al. (2017: 153): molecular phylogeny; Martin et al. (2017: 564): phylogeny; Wang et al. (2017: 1): phylogeny; Yiu (2017: 60): species descriptions, key; Bocak et al. (2018: 2): molecular phylogeny; Fallon et al. (2018: 2, 96): genomes, bioluminiscence; Kusy et al. (2018a: 5): molecular phylogeny; Kusy et al. (2018b: 2): molecular phylogeny; Tan (2018: 127, 135): distribution, photographs; Zhang et al. (2018: 3): molecular phylogeny; Amaral et al. (2019: 283): molecular phylogeny [also as Rhagophtalmidae [sic!]]; Chen et al. (2019: 4): molecular phylogeny; Jeng (2019: 8): biofluorescence, biology; Kundrata et al. (2019: 1259): molecular phylogeny; Martin et al. (2019: 2): molecular phylogeny [also as Rhagophthalminae]; McKenna et al. (2019: 4): molecular phylogeny; Liu et al. (2020: 46): luciferase, phylogeny [also as Rhagophthalminae]; Rosa et al. (2020: 7): molecular phylogeny; Roza (2020: 421): morphology, distribution; Zhang et al. (2020: 1): molecular phylogeny, bioluminescence; Douglas et al. (2021: 2): molecular phylogeny; Ge et al. (2021: 3): mitogenomic phylogeny; Kusy et al. (2021: 111): molecular phylogeny; Li et al. (2021a: 5): remark; Li et al. (2021b: 1): phylogeny, distribution, morphology; Seri and Rahman (2021: 715): remark; Cai et al. (2022: 6): molecular phylogeny; Ge et al. (2022: 2): mitogenomic phylogeny; Powell et al. (2022: 1): molecular phylogeny, bioluminescence [also as Rhagophtalmidae [sic!]]. In addition to the aforementioned literature, Rhagophthalmidae were mentioned in some student works, e.g., PhD theses by Ho (2002), Jeng (2008), and Roza (2022).

#### Remarks.

As defined here, Rhagophthalmidae include 12 genera (one of them with two subgenera) and 66 species distributed primarily in East, South, and Southeast Asia, with a few species found on the border of South and Central Asia (i.e., Afghanistan). Males can be recognized by antennae with 12 antennomeres, with antennomere III longer than antennomere II. In cases where the antennae are serrate or pectinate, antennomere III is not simple, i.e., the serration or rami begin on antennomere III. Females are more (e.g., *Diplocladon* or *Haplocladon*; see Remarks under these genera) or less (e.g., *Rhagophthalmus*) larviform (for more information, see Kawashima et al. 2010). Known larvae are predators of millipedes, similar to larvae of the closely related Phengodidae. Although Rhagophthalmidae were credited by McDermott (1966) to “Olivier, 1902”, we found no evidence of the publication to which McDermott referred, similar to Lawrence and Newton (1995: 858).

### ﻿Genus Bicladodrilus

Taxon classificationAnimaliaColeopteraRhagophthalmidae

Pic, 1921

B0185C71-CABA-51E4-964E-32CACCB767FD

Bicladodrilus Pic, 1921a: 15. Gender: masculine. Type species. Bicladodrilusbakeri Pic, 1921; by monotypy.
Bieladodrilus : Pic 1923: 62 [unavailable name, incorrect subsequent spelling not in prevailing usage].
Bicalodrilus : Pic 1930b: 320 [unavailable name, incorrect subsequent spelling not in prevailing usage].

#### Literature.

Pic (1921a: 15): original description; Pic (1923: 62): species description [as *Bieladodrilus* [sic!]]; Pic (1930b: 320): remark [as *Bicalodrilus* [sic!]], key; Wittmer (1941: 197): catalogue, distribution; Wittmer (1944: 211): catalogue; Bocakova et al. (2007: 484): molecular phylogeny; Hunt et al. (2007: suppl.): molecular phylogeny; Bocak et al. (2008: 2019): molecular phylogeny; Levkanicova and Bocak (2009: 214): molecular phylogeny; Costa and Zaragoza-Caballero (2010: 134): remark; Kawashima et al. (2010: 139): book chapter; Kundrata and Bocak (2011a: 57): remark; Kundrata and Bocak (2011b: 370): molecular phylogeny; Kundrata et al. (2013: 202): molecular phylogeny; Kundrata et al. (2014: 167): molecular phylogeny; Bocak et al. (2016: suppl.): molecular phylogeny; Kovalev and Kirejtshuk (2016: 205): remark; Kundrata et al. (2016: 296): molecular phylogeny; Bocak et al. (2018: 4): molecular phylogeny; Kundrata et al. (2019: 1263): molecular phylogeny; Liu et al. (2020: 46): remark. In addition to the aforementioned literature, this genus was included in PhD theses by Jeng (2008) and Roza (2022).

#### Remarks.

This genus currently contains two described species from the Philippines and Vietnam, respectively. The generic assignment of a specimen reported as “*Bicladodrilus* sp.” from China, which was used in the molecular phylogenetic analyses by Bocakova et al. (2007), Bocak et al. (2008, 2018), Levkanicova and Bocak (2009), and other studies, needs a careful re-examination. *Bicladodrilus* is similar to *Bicladum* and *Diplocladon* in having strongly bipectinate antennae and long elytra. This generic complex is in need of revision.

### ﻿ Bicladodrilus bakeri

Taxon classificationAnimaliaColeopteraRhagophthalmidae

Pic, 1921

0BD39A61-BB56-51D6-993C-E1CC29B97B1B

Bicladodrilus bakeri Pic, 1921a: 15.

#### Type depository.

Described based on an unknown number of specimens. Syntype, male (MNHN).

#### Type locality.

Philippines: Mindanao.

#### Distribution.

Philippines.

#### Literature.

Pic (1921b: 15): original description; Pic (1923: 63): comparison with *B.laticollis* Pic, 1923; Wittmer (1941: 197): catalogue, distribution; Wittmer (1944: 211): catalogue.

### ﻿ Bicladodrilus laticollis

Taxon classificationAnimaliaColeopteraRhagophthalmidae

Pic, 1923

BC39FB9B-2F7E-5312-BA3E-E71FDBA0BD26

Bieladodrilus [sic!] laticollis Pic, 1923: 62.

#### Type depository.

Described based on an unknown number of specimens. Syntype, male (MNHN).

#### Type locality.

Vietnam: Lào Cai [Tonkin: Lao-Kay].

#### Distribution.

Vietnam.

#### Literature.

Pic (1923: 62): original description; Wittmer (1944: 211): catalogue.

### ﻿Genus Bicladum

Taxon classificationAnimaliaColeopteraRhagophthalmidae

Pic, 1921

76BB5109-16CE-560A-925C-0AD76EC685EE

Bicladum Pic, 1921b: 12. Gender: neuter. Type species. Bicladummultipunctatum Pic, 1921; by monotypy.
Bicladon : Pic 1930a: 2 [unavailable name, incorrect subsequent spelling].

#### Literature.

Pic (1921b: 12): original description; Pic (1921a: 15): comparison with *Bicladodrilus*; Pic (1930a: 2): species description [as *Bicladon* [sic!]]; Pic (1930b: 320, 321): remark, key [as *Bicladon* [sic!]]; Wittmer (1944: 211): catalogue [as *Bicladon* [sic!]]; Lawrence et al. (2010b: 175): remark [as *Bicladon* [sic!]]; Kundrata and Bocak (2011a: 57): remark [as *Bicladon* [sic!]]; Janisova and Bocakova (2013: 3): remark [as *Bicladon* [sic!]]; Kovalev and Kirejtshuk (2016: 205): remark [as *Bicladon* [sic!]]. In addition to the aforementioned literature, this genus was included in a PhD thesis by Jeng (2008).

#### Remarks.

This genus currently contains two described species from Borneo and Sumatra, respectively. It is similar to *Bicladodrilus* and *Diplocladon* in having strongly bipectinate antennae and long elytra. This generic complex is in need of revision.

### ﻿ Bicladum mjobergi

Taxon classificationAnimaliaColeopteraRhagophthalmidae

Pic, 1930

31AB7D66-0507-513D-9AAD-818B41A21FD3

Bicladon [sic!] *mjöbergi* [sic!] Pic, 1930a: 2, 4.

#### Type depositories.

Described based on an unknown number of specimens. One syntype, male (MNHN), two syntypes, males (labelled as “Typus” and “Paratypus”) (SMNH).

#### Type locality.

Indonesia: Sumatra, Medan.

#### Distribution.

Indonesia (Sumatra).

#### Literature.

Pic (1930a: 2, 4): original description; Wittmer (1944: 211): catalogue [as *Bicladon* [sic!]].

#### Remarks.

Pic (1930a: 5) also reported an unnamed variety of *B.mjobergi* based on a specimen from Tjinta Radja. This specimen is deposited in SMNH and bears the label “Typus”; however, based on Article 72.4.1. of the Code (ICZN 1999) it should not be considered a part of the type series.

### ﻿ Bicladum multipunctatum

Taxon classificationAnimaliaColeopteraRhagophthalmidae

Pic, 1921

651CD9BF-32F0-595E-B93C-6167BA93EF71

Bicladum multipunctatum Pic, 1921b: 12.

#### Type depository.

Described based on an unknown number of specimens. Syntype, male (MNHN).

#### Type locality.

Borneo (without any further data).

#### Distribution.

Borneo (probably northern region).

#### Literature.

Pic (1921b: 12): original description; Pic (1930a: 5): comparison with *B.mjobergi*; Wittmer (1944: 211): catalogue [as *Bicladon* [sic!]].

### ﻿Genus Dioptoma

Taxon classificationAnimaliaColeopteraRhagophthalmidae

Pascoe, 1860

53D1C3FD-5D9F-5AE5-8197-1E79BE98F47E

Fig. 1A, B

Dioptoma Pascoe, 1860: 118. Gender: feminine. Type species. Dioptomaadamsii Pascoe, 1860; by monotypy.
Diaptoma : Wijekoon et al. 2016: 70 [unavailable name, incorrect subsequent spelling not in prevailing usage].

#### Literature.

Pascoe (1860: 118): original description, drawings of male habitus, head, and antenna; Pascoe (1862: 323): comparison with *Ochotyra*; Gerstaecker (1863: 409): remark; Gemminger (1869: 1647): catalogue; Gorham (1880: 66): remark; Gorham (1881: 63): remark; Olivier (1885: 372): remark; Gorham (1890: 550): catalogue; Gorham (1895: 309): redescription; Sharp (1899: 251): remark; Gorham (1903: 330): distributional note; Olivier (1907: 63): catalogue; Gahan (1908a: xlviii): remark; Gahan (1908b: 205): remark; Olivier (1910: 1): catalogue; Olivier (1912: 467): remark; Morice (1913: cxviii): introduction of a new species attributed to Gahan; Green (1913: 718): male and female morphology, bioluminescence, drawing of male habitus; McDermott (1914: 304): remark; Blair (1915a: 413): bioluminescence; Blair (1915b: 191): bioluminescence; Blair (1915c: 37): bioluminescence, morphology; Gravely (1915: 502): remark; Bugnion (1916: 83): remark; Pic (1916: 8): species description; Lucas (1920: 241): catalogue; Bugnion (1929: 4): remark; Brues (1941: 41): remark; Harvey (1952: 392): remark; Harvey (1955: 19): checklist, bioluminescence; Bess (1956: 25): remark; McDermott (1964: 50): revision; McDermott (1966: 122): catalogue; Mikšić and Mikšić (1966: 32): remark; Lloyd (1971: 101): remark, drawing of male habitus with distribution of luminous organs; Crowson (1972: 52): remark; Paulus (1975: 78): remark; Herring (1978: 471): checklist; Lloyd (1978: 252): remark, drawing of male habitus with distribution of luminous organs; Lloyd (1979: 302): remark; Ohba (1980: 14): remark; Crowson (1981: 314): remark, drawing of male habitus with distribution of luminous organs; Sivinski (1981: 168): remark; Lloyd (1983: 136): remark, bioluminescence; Hoffmann (1984: 230): remark; Herring (1987: 158): checklist; Cicero (1988: 148): remark; Viviani and Bechara (1993: 615): remark; Wittmer and Ohba (1994: 342): remark; Lawrence and Newton (1995: 857): catalogue, remark; Branham (1996: 18): remark; Ohba et al. (1996a: 17): remark; Viviani and Bechara (1997: 389): remark; Sivinski et al. (1998: 29): remark; Kawashima (2000: 131): remark; Branham and Wenzel (2001: 566): phylogeny; O’Keefe (2002: 182): remark; Branham and Wenzel (2003: 5): phylogeny; Li et al. (2008a: 259): remark; Li et al. (2008b: 495): review; Li and Liang (2008: 111): remark; Bogahawatta et al. (2009: 1): remark; Suzuki and Kobayashi (2009: 30): remark; Chen et al. (2010: 196): remark; Kawashima et al. (2010: 135): book chapter; Kundrata and Bocak (2011a: 57): remark; Oba et al. (2011: 777): remark; Wijekoon et al. (2016: 70): checklist [as *Diaptoma* [sic!]]; Liu et al. (2020: 46): remark. In addition to the aforementioned literature, this genus was included in PhD theses by Jeng (2008) and Roza (2022).

#### Remarks.

This genus currently contains two described species from Bangladesh, India, and Sri Lanka. Males are characterized by short antennae and deeply emarginate eyes, each with a smaller upper portion and a larger lower portion (Fig. 1B). Regarding the gender of *Dioptoma*, Pascoe (1860) treated it as feminine and since the name is not a Greek noun, here we follow Pascoe’s decision.

### ﻿ Dioptoma adamsii

Taxon classificationAnimaliaColeopteraRhagophthalmidae

Pascoe, 1860

355E0479-9A71-59C9-9A06-4C7CD9069AC1

Fig. 1A, B

Dioptoma adamsii Pascoe, 1860: 118.
Dioptoma adamsi : Gemminger 1869: 1647 [unavailable name, incorrect subsequent spelling].
Dioptoma greeni Gahan in Morice 1913: cxviii. Synonymized with D.adamsii (as a variety) by McDermott (1966: 122). McDermott (1966) attributed the name D.greeni to Gahan (1908a: xlviii); however, there is no such name in that publication, and we believe that this name first appeared in 1913.
Dioptoma ademsi : Bogahawatta et al. 2009: 1 [unavailable name, incorrect subsequent spelling not in prevailing usage].

#### Type depository.

Holotype of *D.adamsii*, male (NHMUK). 25 syntypes of *D.greeni* (eight males from Maskeliya, eight males and four females from Dikoya, four males and one female from Bogawantalawa; Fig. 1A) (NHMUK).

#### Type locality of *D.adamsii*.

Bangladesh: Dhaka [“India: Dacca”]. Type localities of *D.greeni*. Sri Lanka: Bogawantalawa, Dikoya, and Maskeliya.

#### Distribution.

Bangladesh, India (Karnataka, Kerala, Tamil Nadu, Uttarakhand), Sri Lanka.

#### Literature.

Pascoe (1860: 118): original description, drawings of male habitus, head, and antenna; Gemminger (1869: 1647): catalogue [as *D.adamsi* [sic!]]; Gorham (1880: 66): remark [as *D.adamsi* [sic!]]; Olivier (1885: 372): remark [as *D.adamsi* [sic!]]; Gorham (1890: 550): catalogue [as *D.adamsi* [sic!]]; Gorham (1895: 310): redescription, distributional note [as *D.adamsi* [sic!]]; Sharp (1899: 251): remark [as *D.adamsi* [sic!]]; Gorham (1903: 330): distributional note [as *D.adamsi* [sic!]]; Olivier (1910: 1): catalogue [as *D.adamsi* [sic!]]; Morice (1913: cxviii): original description of *D.greeni* (attributed to Gahan), remarks on *D.adamsi* [sic!]; Green (1913: 718): male and female morphology, bioluminescence, drawing of male habitus [as *D.adamsi* [sic!]]; McDermott (1914: 304): remark; Blair (1915a: 413): bioluminescence [as *D.adamsi* [sic!]]; Blair (1915b: 191): bioluminescence [as *D.adamsi* [sic!]]; Blair (1915c: 37): bioluminescence, morphology [as *D.adamsi* [sic!]]; Gravely (1915: 502): remark [as *D.adamsi* [sic!]]; Bugnion (1916: 96): remark [as *D.adamsi* [sic!]]; Pic (1916: 8): comparison with *D.atripennis* Pic, 1916 [as *D.adamsi* [sic!]]; Lucas (1920: 241): catalogue [as *D.adamsi* [sic!]]; Brues (1941: 41): remark [as *D.adamsi* [sic!]]; Harvey (1952: 450): remark, bioluminescence [as *D.adamsi* [sic!]]; McDermott (1964: 50): redescription [as *D.adamsi* [sic!]]; McDermott (1966: 122): catalogue, synonymization of *D.greeni* with *D.adamsi* [sic!]; Lloyd (1971: 101): remark, drawing of male habitus with distribution of luminous organs [as *D.adamsi* [sic!]]; Lloyd (1978: 252): remark, drawing of male habitus with distribution of luminous organs [as *D.adamsi* [sic!]]; Lloyd (1979: 302): remark [as *D.adamsi* [sic!]]; Crowson (1981: 314): remark, drawing of male habitus with distribution of luminous organs [*as D.adamsi* [sic!]]; Sivinski (1981: 168): remark [as *D.adamsi* [sic!]]; Lloyd (1983: 136): remark, bioluminescence [as *D.adamsi* [sic!]]; Hoffmann (1984: 230): remark [as *D.adamsi* [sic!]]; Branham (1996: 18): remark [as *D.adamsi* [sic!]]; Ohba et al. (1996a: 17): remark; Sivinski et al. (1998: 29): remark [as *D.adamsi* [sic!]]; Kawashima (2000: 131): remark; Branham and Wenzel (2001: 567): phylogeny [as *D.adamsi* [sic!]]; Branham and Wenzel (2003: 5): phylogeny [as *D.adamsi* [sic!]]; Li et al. (2008b: 496): review [also as *D.adamsi* [sic!]]; Bogahawatta et al. (2009: 1): remark [both *D.ademsi* [sic!] and *D.greeni*]; Kawashima et al. (2010: 135): book chapter [as *D.adamsi* [sic!]]; Wijekoon et al. (2016: 70): catalogue [both *Diaptomaadamsi* [sic!] and *D.greeni*]. In addition to the aforementioned literature, this species was included in PhD theses by Jeng (2008) and Roza (2022).

#### Remarks.

This species was referred to as “*adamsi*” in the majority of publications. The original spelling “*adamsii*” was used only by McDermott (1914), Ohba et al. (1996a), and Kawashima (2000). However, following Article 33.4. of the Code (ICZN 1999), the original spelling should be maintained. It should be noted that the current concept of *D.adamsii* may include several species.

### ﻿ Dioptoma atripennis

Taxon classificationAnimaliaColeopteraRhagophthalmidae

Pic, 1916

DB60CEF4-783B-5564-80FC-4CF0FF6A6ED3

Dioptoma atripennis Pic, 1916: 8.

#### Type depository.

Described based on an unknown number of specimens. Two syntypes, males (MNHN).

#### Type locality.

India: Tamil Nadu, Madurai [Madura].

#### Distribution.

India (Tamil Nadu).

#### Literature.

Pic (1916: 8): original description; McDermott (1966: 122): catalogue; Li et al. (2008b: 496): review.

### ﻿Genus Diplocladon

Taxon classificationAnimaliaColeopteraRhagophthalmidae

Gorham, 1883

09D151D2-3825-57C6-A32F-819651C145ED

Fig. 1C

Diplocladon Gorham, 1883a: 5. Gender: neuter. Type species. Diplocladonhasseltii Gorham, 1883, by monotypy.
Diplocadum : Pic 1921b: 12 [unavailable name, incorrect subsequent spelling not in prevailing usage].
Diplocladum : Pic 1928: 86 [unavailable name, incorrect subsequent spelling not in prevailing usage].
Diplocadon : Viviani and Bechara 1993: 615 [unavailable name, incorrect subsequent spelling not in prevailing usage].
Diploclodon : Tan 2018: 135 [unavailable name, incorrect subsequent spelling not in prevailing usage].

#### Literature.

Gorham (1883a: 5): original description; Gorham (1883b: 249, 250): comparison with *Haplocladon*; Gorham (1883c: 597): remark; Gorham (1887: 76): catalogue, redescription; Waterhouse (1890: 25): remark, figure of male habitus; Gorham (1895: 310): remark; Olivier (1910: 8): catalogue; Lucas (1920: 243): catalogue; Rüschkamp (1920: 386): distributional note; Pic (1921b: 12): comparison with *Bicladum* and *Monodrilus* [as *Diplocadum* [sic!]]; Pic (1928: 86): remark [as *Diplocadum* [sic!]]; Pic (1930a: 2): distributional note [as *Diplocladum* [sic!]]; Pic (1930b: 320): remark, key; Ridley (1934: 60): larval biology and morphology; Wittmer (1944: 211): catalogue; Haneda (1950: 2): bioluminescence; Harvey (1952: 451): bioluminescence, drawings of female habitus with position of luminous organs, photographs of male and female habitus; Crowson (1955: 68, 171): remark; Haneda (1955: 364): remark, bioluminescence; Harvey (1955: 19): checklist, bioluminescence; Harvey (1957: 554): remark; McDermott (1964: 50): remark; Nakane (1968: 3): remark; Lloyd (1971: 101): remark, drawing of female habitus with luminous organs; Crowson (1972: 52): remark; Paulus (1972: 49): remark; Halverson et al. (1973: 1332): biology, bioluminescence; McElroy et al. (1974: 417): remark; Paulus (1975: 78): remark; Case and Strause (1978: 332): remark; Herring (1978: 471): checklist; Lloyd (1978: 252): remark, drawing of female habitus with distribution of luminous organs; Ohba (1980: 14): remark; Crowson (1981: 314): remark, drawing of female habitus with distribution of luminous organs; Sivinski (1981: 168): remark; Lloyd (1983: 136): remark, bioluminescence; Hoffmann (1984: 229): remark; Haneda (1985: 167): bioluminescence; Herring (1987: 157): checklist; Cicero (1988: 148): remark; De Keyzer (1989: 54): remark; Viviani and Bechara (1993: 615): remark [as *Diplocadon* [sic!]]; Wittmer and Ohba (1994: 350): remark; Lawrence and Newton (1995: 857): catalogue, remark; Branham (1996: 18): remark; Ohba et al. (1996a: 13): remark; Ohba et al. (1996b: 30): remark; Ohba (1997a: 17): remark; Viviani and Bechara (1997: 389): remark [as *Diplocadon* [sic!]]; Branham and Wenzel (2001: 566): phylogeny; O‘Keefe (2002: 182): remark; Branham and Wenzel (2003: 3): remark; Li and Liang (2008: 109): remark, female description; Li et al. (2008b: 495): review; Suzuki and Kobayashi (2009: 31): remark; Kawashima et al. (2010: 135): book chapter, figures of male and female habitus, and details of female abdominal segments; Kundrata and Bocak (2011a: 57): remark; Oba et al. (2011: 777): remark; Yiu (2012: 30): catalogue, figures of habitus; Yiu (2013: 113): remark, biology; Kovalev and Kirejtshuk (2016: 205): remark; Yiu (2017: 64): description of a new species, identification key; Tan (2018: 135): possible larva, distribution, figures of larval habitus and bioluminescence [also as *Diploclodon*]; Liu et al. (2020: 46): remark; Lawrence et al. (2021: 456): wing morphology; Li et al. (2021b: 4): remark; Seri and Rahman (2021: 721): remark. In addition to the aforementioned literature, this genus was included in PhD theses by Jeng (2008) and Roza (2022).

#### Remarks.

See more information on *Haplocladon*, which was once considered a subgenus of *Diplocladon* (Gorham 1883b) or even its synonym (e.g., Wittmer 1944), under the genus name *Haplocladon* below. Some authors who mentioned *Diplocladon* were actually probably referring to *Haplocladon* (for more details, see Remarks under *D.hasseltii*). *Diplocladon* currently contains two described species, one from China and one from Indonesia. It is similar to *Bicladodrilus* and *Bicladum* in having strongly bipectinate antennae (Fig. 1C) and long elytra. This generic complex is in need of revision.

### ﻿ Diplocladon atripenne

Taxon classificationAnimaliaColeopteraRhagophthalmidae

Yiu, 2017

BE33CF86-A240-5027-AE5B-84EC4C7ED7A1

Diplocladon atripennis [sic!] Yiu, 2017: 64.

#### Type depository.

Holotype, male (TLES). Paratype, male (TLES).

#### Type locality.

China: Hong Kong, Lantau, Wo Tin (22.27351°N, 113.98819°E).

#### Distribution.

China (Hong Kong).

#### Literature.

Yiu (2017: 64): original description, figures of male habitus, pregenital segments and genitalia.

### ﻿ Diplocladon hasseltii hasseltii

Taxon classificationAnimaliaColeopteraRhagophthalmidae

Gorham, 1883

775AC322-4459-51C5-8356-43D6D4CC8FE2

Fig. 1C

Diplocladon hasseltii Gorham, 1883a: 6.
Diplocladon hasselti : Olivier 1910: 8 [unavailable name, incorrect subsequent spelling not in prevailing usage].

#### Type depository.

Described based on two specimens (Gorham 1887). One syntype, male (RMNH); one syntype, male (MNHN).

#### Type locality.

Indonesia: Sumatra, Boenga mas (Palembang).

#### Distribution.

Indonesia (Sumatra, Java).

#### Literature.

Gorham (1883a: 6): original description; Gorham (1887: 76): catalogue, redescription; Waterhouse (1890: 25): remark, figure of male habitus; Olivier (1910: 8): catalogue [as *D.hasselti* [sic!]]; Lucas (1920: 243): catalogue [as *D.hasselti* [sic!]]; Ridley (1934: 60): larval biology and morphology [as *D.hasselti* [sic!]]; Wittmer (1944: 211): catalogue [as *D.hasselti* [sic!]]; Haneda (1950: 2): bioluminescence, drawings of adult male and female, and position of luminous organs; Harvey (1952: 451): bioluminescence, drawings of female habitus with position of luminous organs, photographs of male and female habitus; Haneda (1955: 364): remark, bioluminescence; Lloyd (1971: 101): remark, drawing of female habitus with luminous organs [as *D.hasselti* [sic!]]; Lloyd (1978: 252): remark, drawing of female habitus with distribution of luminous organs [as *D.hasselti* [sic!]]; Crowson (1981: 314): remark, drawing of female habitus with distribution of luminous organs [*as D.hasselti* [sic!]]; Sivinski (1981: 168): remark [as *D.hasselti* [sic!]]; Lloyd (1983: 136): remark, bioluminescence [as *D.hasselti* [sic!]]; Hoffmann (1984: 229): remark [as *D.hasselti* [sic!]]; Haneda (1985: 167): bioluminescence, drawings of adult male and female, and position of luminous organs [*as D.hasselti* [sic!]]; De Keyzer (1989: 54): remark [as *D.hasselti* [sic!]]; Wittmer and Ohba (1994: 350): remark [as *D.hasselti* [sic!]]; Branham (1996: 18): remark [as *D.hasselti* [sic!]]; Ohba et al. (1996a: 13): remark; Ohba et al. (1996b: 30): remark; Ohba (1997a: 17): remark; Li and Liang (2008: 109): remark; Kawashima et al. (2010: 135): book chapter, figures of male and female habitus, and details of female abdominal segments [as *D.hasselti* [sic!]]; Yiu (2017: 64): comparison with *D.atripennis*; Lawrence et al. (2021: 456): wing morphology, figure of hind wing [as *D.hasselti* [sic!]]. In addition to the aforementioned literature, this species was included in PhD theses by Jeng (2008) and Roza (2022).

#### Remarks.

Based on the available figures, adults of both sexes which were reported by Haneda (1950) from Singapore, and repeatedly mentioned in subsequent studies (e.g., Harvey 1952; Haneda 1955, 1985; Lloyd 1971, 1978; Crowson 1981; Kawashima et al. 2010), are probably members of *Haplocladon*. We are aware of several *Haplocladon* specimens from Singapore (deposited in NHMUK) but no *Diplocladon* are known from that area.

### ﻿ Diplocladon hasseltii testaceum

Taxon classificationAnimaliaColeopteraRhagophthalmidae

Pic, 1921

66186E05-2202-5317-A540-5D6307EE2409

Diplocadum [sic!] hasselti [sic!] var.testaceum Pic, 1921b: 12.
Diplocladum [sic!] hasselti [sic!] var.testaceus [sic!]: Pic 1930a: 2.

#### Type depository.

Described based on an unknown number of specimens. No type specimen found in MNHN by RK.

#### Type locality.

Indonesia: Sumatra.

#### Distribution.

Indonesia (Sumatra).

#### Literature.

Pic (1921a: 12): original description [as a variety of *Diplocadum* [sic!] *hasselti* [sic!]]; Pic (1930b: 2): distributional note; Wittmer (1944: 211): catalogue.

#### Remarks.

The name “*testaceum*” is deemed to be subspecific according to Article 45.6.4. of the Code (ICZN 1999).

### ﻿Genus Dodecatoma

Taxon classificationAnimaliaColeopteraRhagophthalmidae

Westwood, 1849

7523762A-0AAD-572B-B413-AF186157C9EC

Dodecatoma Westwood, 1849: 1. Gender: feminine. Type species. Dodecatomabicolor Westwood, 1849, by monotypy.
Dodecatomax : Crowson 1955: 171 [unavailable name, incorrect subsequent spelling not in prevailing usage].

#### Literature.

Westwood (1849: 1): original description, drawings of male habitus, head, mouthparts, antenna, and leg; Schaum (1850: 165): morphology, remark; Lacordaire (1857: 377): catalogue, redescription; Motschulsky (1861: 134): comparison with *Pachytarsus* Motschulsky, 1861; Gemminger (1869: 1686): catalogue; Gorham (1895: 309): species description, remark; Olivier (1910: 8): catalogue; Fowler (1912: 138): remark; Lucas (1920: 246): catalogue; Rüschkamp (1920: 386): distributional note; Pic (1921b: 12): species description; Pic (1924: 713): species description, remark; Pic (1930b: 321): remark; Wittmer (1941: 197): catalogue; Wittmer (1944: 211): catalogue; Harvey (1952: 392): remark; Crowson (1955: 68, 171): remark [also as *Dodecatomax* [sic!]]; Goidanich (1957: 565): remark; McDermott (1964: 50): remark; Paulus (1972: 49): remark; Wittmer (1979: 89): species description, drawing of male antenna; Lawrence and Newton (1995: 857): catalogue, remark; Wittmer (1995: 110): species descriptions; Bocak (2007: 225): catalogue; Li et al. (2008b: 495): review; Kawashima et al. (2010: 135): book chapter; Kundrata and Bocak (2011a: 58): remark; Oba et al. (2011: 777): remark; Kazantsev (2012: 349): descriptions of new species, identification key; Johnson et al. (2012: 178): ICZN case; ICZN (2014: 195): ICZN case; Kovalev and Kirejtshuk (2016: 205): remark; Liu et al. (2020: 46): remark; Lawrence et al. (2021: 456): wing morphology. In addition to the aforementioned literature, this genus was included in PhD theses by Jeng (2008) and Roza (2022).

#### Remarks.

*Dodecatoma* currently contains eight described species from Afghanistan, India, Nepal, Indonesia, and the Philippines. This genus is in need of revision; taxa from Southeast Asia should be removed from *Dodecatoma*, and the generic assignment of the species with serrate antennae described recently by Kazantsev (2012) needs careful re-examination (the remaining species of *Dodecatoma*, including the type species, have pectinate antennae).

### ﻿ Dodecatoma bicolor

Taxon classificationAnimaliaColeopteraRhagophthalmidae

Westwood, 1849

E8B995FD-B4FD-5C72-B0DF-1950C9E6E19B

Dodecatoma bicolor Westwood, 1849: 1.

#### Type depository.

Described based on an unknown number of specimens. Syntype, male (OUMNH).

#### Type locality.

India: Deccan Plateau (without further details; “North India” on the label of the syntype in OUMNH).

#### Distribution.

India (Karnataka, Maharashtra).

#### Literature.

Westwood (1849: 1): original description, drawings of male habitus and body parts; Schaum (1850: 165): morphology, remark; Gemminger (1869: 1686): catalogue; Gorham (1895: 309): distributional note; Olivier (1910: 8): catalogue; Lucas (1920: 246): catalogue; Pic (1924: 714): comparison with *D.testaceiceps* Pic, 1924; Pic (1930b: 321): remark; Wittmer (1944: 211): catalogue; Lawrence and Newton (1995: 858): catalogue, remark; Bocak (2007: 225): catalogue; Johnson et al. (2012: 178): ICZN case; ICZN (2014: 195): ICZN case; Lawrence et al. (2021: 456): wing morphology, figure of hind wing. In addition to the aforementioned literature, this species was included in PhD theses by Jeng (2008) and Roza (2022).

### ﻿ Dodecatoma fuscicornis fuscicornis

Taxon classificationAnimaliaColeopteraRhagophthalmidae

Gorham, 1895

4B374DE5-B946-5D8B-95B0-DFC9FB3DB09F

Dodecatoma fuscicornis Gorham, 1895: 309.

#### Type depository.

Described based on “several examples” (Gorham 1895: 309). Three syntypes, males (NHMUK). Several specimens from Belgaum deposited in MNHN are potentially syntypes (RK pers. obs.).

#### Type locality.

India: Karnataka, Belgaum.

#### Distribution.

India (Karnataka).

#### Literature.

Gorham (1895: 309): original description; Olivier (1910: 8): catalogue; Wittmer (1944: 211): catalogue; Wittmer (1979: 90): comparison with other species; Johnson et al. (2012: 179): ICZN case.

### ﻿ Dodecatoma fuscicornis testaceicornis

Taxon classificationAnimaliaColeopteraRhagophthalmidae

Pic, 1921

661DC4F8-8805-52CF-B0ED-22B10DE19E3F

Dodecatoma fuscicornis var. testaceicornis Pic, 1921b: 12.

#### Type depository.

Described based on an unknown number of specimens. Syntype, male (MNHN).

#### Type locality.

Indonesia: Java.

#### Distribution.

Indonesia (Java).

#### Literature.

Pic (1921b: 12): original description; Wittmer (1944: 212): catalogue.

#### Remarks.

The name “*testaceicornis*” is deemed to be subspecific according to Art. 45.6.4. of the Code (ICZN 1999). This taxon is not morphologically similar to *D.fuscicornis* Gorham, 1895 nor to any other species of *Dodecatoma*.

### ﻿ Dodecatoma gracilis

Taxon classificationAnimaliaColeopteraRhagophthalmidae

Wittmer, 1995

330A1435-D4AC-5FE7-935B-AB09FC2839CC

Dodecatoma gracilis Wittmer, 1995: 110.

#### Type depository.

Holotype, male (NHMB). One paratype, male (NHMB).

#### Type locality.

Nepal: near Simra Abhabar, 200 m.

#### Distribution.

Nepal.

#### Literature.

Wittmer (1995: 110): original description, figures of male antenna and genitalia; Bocak (2007: 225): catalogue; Johnson et al. (2012: 179): ICZN case; Kazantsev (2012: 349): comparison with *D.saluki* and *D.schmidti*, identification key.

### ﻿ Dodecatoma parvicornis

Taxon classificationAnimaliaColeopteraRhagophthalmidae

Wittmer, 1979

289E71E3-C000-5BC8-A15F-DC5C23F3E277

Dodecatoma parvicornis Wittmer, 1979: 89.

#### Type depository.

Holotype, male (NHMB). Two paratypes, males (NHMB).

#### Type locality.

Afghanistan: Nuristan, Baschgultal.

#### Distribution.

Afghanistan, Pakistan.

#### Literature.

Wittmer (1979: 89): original description, drawing of antenna; Bocak (2007: 225): catalogue; Johnson et al. (2012: 179): ICZN case.

### ﻿ Dodecatoma riedeli

Taxon classificationAnimaliaColeopteraRhagophthalmidae

Wittmer, 1995

C9E0D1A8-DCC2-5BAA-8FA8-525AE1DB9477

Dodecatoma riedeli Wittmer, 1995: 112.

#### Type depository.

Holotype, male (SMNS). Three paratypes, males (NHMB).

#### Type locality.

India: Uttarakhand [“Uttar Pradesh”], Rishikesh.

#### Distribution.

India (Uttarakhand).

#### Literature.

Wittmer (1995: 112): original description, figures of male antenna and genitalia; Bocak (2007: 225): catalogue; Johnson et al. (2012: 179): ICZN case; Kazantsev (2012: 349): comparison with *D.saluki* and *D.schmidti*, identification key.

### ﻿ Dodecatoma saluki

Taxon classificationAnimaliaColeopteraRhagophthalmidae

Kazantsev, 2012

CCA99148-96F4-5779-BE25-25814566734A

Dodecatoma saluki Kazantsev, 2012: 349.

#### Type depository.

Holotype, male (ICM). One paratype, male (NKME).

#### Type locality.

India: Uttarakhand [Uttaranchal], Nainital Distr., 5 km SE Mukteshwar, Satkhol.

#### Distribution.

India (Uttarakhand), Nepal.

#### Literature.

Kazantsev (2012: 349): original description, figures of male habitus, pregenital segments, and genitalia.

### ﻿ Dodecatoma schmidti

Taxon classificationAnimaliaColeopteraRhagophthalmidae

Kazantsev, 2012

F99B3A22-23E1-58EA-804B-087B98DA69F4

Dodecatoma schmidti Kazantsev, 2012: 349.

#### Type depository.

Holotype, male (NKME).

#### Type locality.

Nepal: Kali Gandaki valley, Upper Lete.

#### Distribution.

Nepal.

#### Literature.

Kazantsev (2012: 349): original description, drawings of male basal antennomeres and genitalia.

### ﻿ Dodecatoma testaceiceps

Taxon classificationAnimaliaColeopteraRhagophthalmidae

Pic, 1924

76678D3E-3F2D-5EF5-960E-7436140C45C8

Dodecatoma testaceiceps Pic, 1924: 713.
Dodecatoma testaceipes : Wittmer 1944: 212 [unavailable name, incorrect subsequent spelling not in prevailing usage].

#### Type depository.

Described based on an unknown number of specimens (but probably only one). One syntype, male (MNHN).

#### Type locality.

Philippines: Luzon, Mt. Maquiling.

#### Distribution.

Philippines.

#### Literature.

Pic (1924: 713): original description; Wittmer (1941: 197): catalogue; Wittmer (1944: 212): catalogue [as *D.testaceipes* [sic!]].

#### Remarks.

This species clearly does not represent a member of Rhagophthalmidae and needs to be transferred into a proper family in a future revision.

### ﻿Genus Falsophrixothrix

Taxon classificationAnimaliaColeopteraRhagophthalmidae

Pic, 1937

7D56A634-B479-5222-9EA0-68DFEE97FAEE

Fig. 1D

Falsophrixothrix Pic, 1937: 138. Gender: feminine. Type species. Phrixothrixjavanus [sic!] Pic, 1914; by original designation (Pic 1937: 138).

#### Literature.

Olivier (1911: 20): species description [as *Phrixothrix*]; Pic (1914: 13): species description [as *Phrixothrix*]; Pic (1921a: 16): species description [as *Phrixothrix*]; Pic (1937: 138): original generic description; Wittmer (1938: 301): description of an aberration [term used to denote a class of individuals within a species; unavailable name; see Glossary in ICZN (1999)]; Wittmer (1939: 23): species description; Wittmer (1944: 217): catalogue; Pic (1951: 5): species description; Crowson (1972: 52): remark; Paulus (1975: 78): remark; Herring (1978: 471): checklist; Herring (1987: 157): checklist; Viviani and Bechara (1993: 615): remark; Lawrence and Newton (1995: 857): catalogue, remark; Viviani and Bechara (1997: 389): remark; O‘Keefe (2002: 182): remark; Li et al. (2008b: 495): review; Kawashima et al. (2010: 139): book chapter; Lawrence et al. (2010b: 175): remark; Kundrata and Bocak (2011a: 57): remark; Oba et al. (2011: 777): remark; Janisova and Bocakova (2013: 3): remark; Kovalev and Kirejtshuk (2016: 205): remark; Kundrata et al. (2019: 1263): molecular phylogeny; Douglas et al. (2021: 2): molecular phylogeny. In addition to the aforementioned literature, this genus was included in PhD theses by Jeng (2008) and Roza (2022).

#### Remarks.

*Falsophrixothrix* currently contains six described and several undescribed species from Southeast Asia. It can be recognized by its small body size, strongly bipectinate antennae (Fig. 1D), and usually shortened elytra which do not cover the entire abdomen. It should be noted that all previous authors treated the gender of *Falsophrixothrix* as masculine; however, -*thrix* (hair in Greek) is feminine.

### ﻿ Falsophrixothrix costata

Taxon classificationAnimaliaColeopteraRhagophthalmidae

Pic, 1951

BA0BB5FF-34D5-5AA2-9C55-4849D168BF3E

Falsophrixothrix costatus [sic!] Pic, 1951: 5.

#### Type depository.

Described based on an unknown number of specimens. Syntype, male (MNHN).

#### Type locality.

Vietnam: Ho Chi Minh City [Saigon].

#### Distribution.

Vietnam.

#### Literature.

Pic (1951: 5): original description.

### ﻿ Falsophrixothrix flava

Taxon classificationAnimaliaColeopteraRhagophthalmidae

Wittmer, 1939

BB72D281-72BD-5DA6-8A28-163F3AB82B7A

Falsophrixothrix flavus [sic!] Wittmer, 1939: 23.

#### Type depository.

Described based on two specimens. Holotype, male (NHMB); paratype, male (?MZB; in Drescher coll. according to the original description).

#### Type locality.

Indonesia: Java, Parahyangan (= Priangan, Preanger), Tangkuban Perahu [G. Tangkoeban Prahoe].

#### Distribution.

Indonesia (Java).

#### Literature.

Wittmer (1939: 23): original description; Wittmer (1944: 217): catalogue.

### ﻿ Falsophrixothrix humeralis

Taxon classificationAnimaliaColeopteraRhagophthalmidae

Pic, 1937

C75568F4-C11D-547A-A137-0243FBD502C6

Falsophrixothrix humeralis Pic, 1937: 138.
Falsophrixothrix humeralis ab. unicolor Wittmer, 1938: 301 [unavailable name, ICZN 1999].

#### Type depository.

Described based on an unknown number of specimens. Syntype (labelled as “Holotypus”), male (NHMB).

#### Type locality.

Indonesia: Java, Parahyangan (= Priangan, Preanger), Tangkuban Perahu [G. Tangkoeban Prahoe] [only “Java” in the original description, remaining information taken from the locality label under the syntype].

#### Distribution.

Indonesia (Java).

#### Literature.

Pic (1937: 138): original description; Wittmer (1938: 301): description of F.humeralis ab. unicolor; Wittmer (1939: 24): comparison with *F.flavus* [sic!]; Wittmer (1944: 217): catalogue; Pic (1951: 5): comparison with *F.costatus* [sic!]. In addition to the aforementioned literature, this species was included in a PhD thesis by Jeng (2008).

#### Remarks.

Wittmer (1938: 301) described the aberration of *F.humeralis* (ab. unicolor) from Tangkuban Perahu [“G. Tangkoeban Prahoe”] based on material from the collection of F. C. Drescher (possibly in MZB); however, this name is deemed to be infrasubspecific according to the Code (ICZN 1999, Article 45.6.2.).

### ﻿ Falsophrixothrix javana

Taxon classificationAnimaliaColeopteraRhagophthalmidae

(Pic, 1914)

B3DC8E75-AE47-57D9-86F2-DC1F022D5A39

Phrixothrix javanus [sic!] Pic, 1914: 13.
Falsophrixothrix javanus [sic!]: Pic 1937: 138.

#### Type depository.

Described based on an unknown number of specimens. Syntype, male (MNHN).

#### Type locality.

Indonesia: Java.

#### Distribution.

Indonesia (Java).

#### Literature.

Pic (1914: 13): original description [as *Phrixothrix*]; Pic (1921a: 16): comparison with *F.punctatus* [sic!] (Pic, 1921) [as *Phrixothrix*]; Pic (1937: 138): comparison with *F.humeralis*; Wittmer (1939: 24): comparison with *F.flavus* [sic!]; Wittmer (1944: 217): catalogue.

### ﻿ Falsophrixothrix punctata

Taxon classificationAnimaliaColeopteraRhagophthalmidae

(Pic, 1921)

F5F99FE0-E573-55B0-B6C8-C9CE52DA4F6A

Phrixothrix punctatus [sic!] Pic, 1921a: 16.
Falsophrixothrix punctatus [sic!]: Wittmer 1944: 217.

#### Type depository.

Described based on an unknown number of specimens. Syntype, male (MNHN).

#### Type locality.

Singapore.

#### Distribution.

Singapore.

#### Literature.

Pic (1921a: 16): original description [as *Phrixothrix*]; Wittmer (1944: 217): catalogue.

### ﻿ Falsophrixothrix pygmaea

Taxon classificationAnimaliaColeopteraRhagophthalmidae

(Olivier, 1911)

59B93D8D-12EB-5D5A-885B-DADB41FE9500

Phrixothrix pygmaeus [sic!] E. Olivier, 1911: 19.
Falsophrixothrix pygmaeus [sic!]: Wittmer 1939: 24.

#### Type depository.

Described based on an unknown number of specimens. At least one syntype, male (RMNH). Five additional male specimens in RMNH (originally from the Zoological Museum, Amsterdam, ZMAN) may also be syntypes (RK pers. obs.).

#### Type locality.

Indonesia, Java: Banyuwangi [Banjoewangi].

#### Distribution.

Indonesia (Java).

#### Literature.

Olivier (1911: 19): original description [as *Phrixothrix*]; Wittmer (1939: 24): comparison with *F.flavus* [sic!]; Wittmer (1944: 217): catalogue.

### ﻿Genus Haplocladon

Taxon classificationAnimaliaColeopteraRhagophthalmidae

Gorham, 1883

EAF9466D-67F3-565B-8A06-24E3B422EDC4

Haplocladon Gorham, 1883b: 249 [as a subgenus of Diplocladon Gorham, 1883]. Gender: neuter. Type species. Haplocladongorhami Kundrata, 2022, nom. nov. [replacement name for Diplocladonhasseltii Gorham, 1883b]; by monotypy.

#### Literature.

Gorham (1883b: 249): original description; Gorham (1895: 310): remark; Gorham (1903: 330): species description; Olivier (1910: 8): catalogue; Wittmer (1944: 211): catalogue; Crowson (1955: 68): remark; Paulus (1972: 49): remark; Li and Liang (2008: 109): remark [as *D.haplocladon* [sic!]]. In addition to the aforementioned literature, this genus was included in a PhD thesis by Jeng (2008).

#### Remarks.

Gorham (1883b) originally described *Haplocladon* as a subgenus of *Diplocladon* but later treated it as a separate genus (Gorham 1895, 1903). Unfortunately, he named type species of both *Diplocladon* and *Haplocladon* as “*hasseltii*” (Gorham 1883a, b), which probably confused some subsequent authors who treated *Haplocladon* as a synonym of *Diplocladon* (Olivier 1910; Wittmer 1944; Li and Liang 2008). Crowson (1955) and Paulus (1972) again considered *Haplocladon* a separate genus. Since *Haplocladon* differs at first sight from *Diplocladon* by the unipectinate antennae (versus bipectinate in *Diplocladon*), we prefer to keep *Haplocladon* at a generic level. Because *Diplocladonhasseltii* Gorham, 1883a and *Diplocladonhasseltii* Gorham, 1883b (described in subgenus Haplocladon) are primary homonyms, the latter junior name is permanently invalid (Art. 57.2 of the Code; ICZN 1999) and should be replaced by a new name (see below). Currently, *Haplocladon* contains two species, one from Indonesia and one from southern India. Based on the available figures, specimens reported by Haneda (1950) from Singapore and identified as *Diplocladonhasseltii*, which were later mentioned by other authors (e.g., Harvey 1952; Haneda 1955; Lloyd 1971; Lloyd 1978; Crowson 1981; Haneda 1985; Kawashima et al. 2010), are probably members of *Haplocladon*.

### ﻿ Haplocladon gorhami

Taxon classificationAnimaliaColeopteraRhagophthalmidae

Kundrata nom. nov.

2DB5AC3A-A0E0-56B0-BB74-4998C7E54BC8

Diplocladon hasseltii Gorham, 1883b: 250 (described in subgenus Haplocladon). Preoccupied by Diplocladonhasseltii Gorham, 1883a: 6.
Haplocladon haselti : Gorham 1903: 330 [unavailable name, incorrect subsequent spelling not in prevailing usage].

#### Type depository.

Described based on an unknown number of specimens. Two syntypes, males (one from Sumatra, one from Java) (RMNH).

#### Type locality.

Indonesia: Sumatra, Lampung, Soekadana; Java, Batavia.

#### Distribution.

Indonesia (Sumatra, Java).

#### Literature.

Gorham (1883b: 250): original description; Gorham (1903: 330): remark [as *H.haselti* [sic!]]; Olivier (1910: 8): catalogue [as *D.hasselti*]; Wittmer (1944: 211): catalogue [as *D.hasselti*].

#### Remarks.

Gorham (1883b: 250) also reported an unnamed variety of *H.hasseltii* as “var. totum testaceum” (i.e., colour description but not the official name of the variety) from Ardjoeno and Batavia in Java. At least one specimen from Batavia labelled as “var.” is present in MNHN. Two specimens from Ardjoeno and one specimen from Batavia deposited in RMNH bear the label “Type”; however, based on Article 72.4.1. of the Code (ICZN 1999), they should not be considered a part of the type series for *Haplocladongorhami*.

### ﻿ Haplocladon indicum

Taxon classificationAnimaliaColeopteraRhagophthalmidae

Gorham, 1903

684F1691-F8CD-5669-A2A3-38C48B6B9D97

Haplocladon indicum Gorham, 1903: 330.
Diplocladon indicum : Olivier 1910: 8.

#### Type depository.

Holotype, male (MNHN).

#### Type locality.

India: Nilgiri Hills.

#### Distribution.

India (Nilgiri Hills).

#### Literature.

Gorham (1903: 330): original description; Olivier (1910: 8): catalogue [as *D.indicum*]; Wittmer (1944: 211): remark [as *D.indicum*]; Li and Liang (2008: 109): remark [as *D.haplocladonindicum* [sic!]]; Yiu (2017: 64): comparison with *Diplocladonatripennis* [sic!] [as *D.indicum*].

### ﻿Genus Menghuoius

Taxon classificationAnimaliaColeopteraRhagophthalmidae

Kawashima, 2000

3074AEFA-C43E-53EA-9545-A062C81DFCED

Menghuoius Kawashima, 2000: 132. Gender: masculine. Type species. Rhagophthalmusingens Fairmaire, 1896, by original designation.
Menghouius : Bocak 2007: 225 [unavailable name, incorrect subsequent spelling not in prevailing usage].
Menhuoius : Li et al. 2008a: 264 [unavailable name, incorrect subsequent spelling not in prevailing usage].

#### Distribution.

China (Anhui, Guangxi, ?Hong Kong, Yunnan, Zhejiang), Myanmar, Vietnam.

#### Literature.

Kawashima (2000: 132): original description; Kawashima (2002: 487): species description, figures of habitus, body parts, and male genitalia; Bocak (2007: 225): catalogue [as *Menghouius* [sic!]]; Li et al. (2008a: 264): distribution, morphology, biology, figures of male antenna and genitalia, and larval and female habitus [as *Menghouius* [sic!]]; Li et al. (2008b: 495): review; Kawashima et al. (2010: 136): book chapter, drawings of head, tarsi, and antenna; Chen et al. (2019: 3): molecular phylogeny; Liu et al. (2020: 46a): luciferase, phylogeny, figures of male and female habitus, and female bioluminescence. In addition to the aforementioned literature, this genus was included in a PhD thesis by Jeng (2008).

#### Remarks.

*Menghuoius* currently contains three described species from China, Myanmar, and Vietnam. It is similar to *Rhagophthalmus* in habitus, short, serrate antennae, and deeply emarginate eyes but differs in the large size and robust mandibles (Kawashima 2000). *Menghuoius* was implicitly considered a junior synonym of *Rhagophthalmus* by Li et al. (2008a) based on the similar morphology of females of both genera. However, since the morphology of highly paedomorphic, larva-like females of Rhagophthalmidae is much less informative than the morphology of adult males, we consider *Menghuoius* a separate genus until a detailed revision of *Rhagophthalmus* and related genera is carried out.

### ﻿ Menghuoius giganteus

Taxon classificationAnimaliaColeopteraRhagophthalmidae

(Fairmaire, 1888)

B7212A60-A337-5C85-96F3-09FF095A9050

Rhagophthalmus giganteus Fairmaire, 1888: 25.
Menghuoius giganteus : Kawashima 2000: 139.
Rhagophthalmus gigantus : Moreira et al. 2022: 7 [unavailable name, incorrect subsequent spelling not in prevailing usage; page number may be changed when the publication is printed].

#### Type depository.

Described based on an unknown number of specimens. Syntype, male (MNHN).

#### Type locality.

China: Yunnan.

#### Distribution.

China (Anhui, Guangxi, Yunnan, Zhejiang).

#### Literature.

Fairmaire (1888: 25): original description [as *R.giganteus*]; Fairmaire (1896: 227): comparison with *R.ingens* [as *R.giganteus*]; Olivier (1902: 88): catalogue [as *R.giganteus*]; Olivier (1910: 1): catalogue [as *R.giganteus*]; Jakobson (1911: 687): catalogue [as *R.giganteus*]; Olivier (1912: 469): revision [as *R.giganteus*]; Winkler (1925: 522): catalogue [as *R.giganteus*]; Wu (1937: 385): catalogue [as *R.giganteus*]; McDermott (1966: 122): catalogue [as *R.giganteus*]; Kawashima (2000: 139): comparison with *R.ingens* [as *R.giganteus*]; Hua (2002: 71): catalogue [as *R.giganteus*]; Bocak (2007: 225): catalogue; Li et al. (2008a: 264): distribution, morphology, biology, figures of male antenna and genitalia, and larval and female habitus [also as *R.giganteus*]; Li et al. (2008b: 496): review [as *R.giganteus*]; Chen et al. (2019: 3): molecular phylogeny; Liu et al. (2020: 46a): luciferase, phylogeny, figures of male and female habitus, and female bioluminescence; Moreira et al. (2022: 7): luciferase, molecular phylogeny [as *R.gigantus* [sic!]]. In addition to the aforementioned literature, this species was included in a PhD thesis by Jeng (2008).

### ﻿ Menghuoius ingens

Taxon classificationAnimaliaColeopteraRhagophthalmidae

(Fairmaire, 1896)

709374AC-B1BB-5FEC-B833-F11691B0E094

Rhagophthalmus ingens Fairmaire, 1896: 227.
Menghuoius ingens : Kawashima 2000: 134.

#### Type depository.

Described based on an unknown number of specimens. Syntype, male (MNHN).

#### Type locality.

China: probably Hong Kong (Fairmaire 1896).

#### Distribution.

China (?Hong Kong), Vietnam.

#### Literature.

Fairmaire (1896: 227): original description [as *R.ingens*]; Olivier (1902: 88): catalogue [as *R.ingens*]; Olivier (1910: 1): catalogue [as *R.ingens*]; Jakobson (1911: 687): catalogue [as *R.ingens*]; Olivier (1912: 469): revision [as *R.ingens*]; Winkler (1925: 522): catalogue [as *R.ingens*]; Wu (1937: 385): catalogue [as *R.ingens*]; McDermott (1966: 122): catalogue [as *R.ingens*]; Kawashima (2000: 134): redescription; Hua (2002: 71): catalogue [as *R.ingens*]; Kawashima (2002: 491): comparison with *M.kusakabei*; Bocak (2007: 225): catalogue [also as *R.ingens*]; Li et al. (2008a: 264): remark, distribution [also as *R.ingens*]; Li et al. (2008b: 496): review [as *R.ingens*]; Kawashima et al. (2010: 136): book chapter, drawings of head, tarsi and antenna; Yiu (2017: 60): comparison with *R.motschulskyi* [as *R.ingens*]; Chen et al. (2019: 11): molecular phylogeny; Liu et al. (2020: 47): remark. In addition to the aforementioned literature, this species was included in a PhD thesis by Jeng (2008).

#### Remarks.

Olivier (1912) mentioned that *R.ingens* could be conspecific with *R.giganteus*.

### ﻿ Menghuoius kusakabei

Taxon classificationAnimaliaColeopteraRhagophthalmidae

Kawashima, 2002

58188A76-AC6C-5932-9346-8AF65C685E1F

Menghuoius kusakabei Kawashima, 2002: 487.

#### Type depository.

Holotype, male (NWU). Four paratypes, males (PCIK).

#### Type locality.

Myanmar: Chin state, Natma Taung National Park near Kanpetlet, Mt. Victoria, ca. 2000 m.

#### Distribution.

Myanmar.

#### Literature.

Kawashima (2002: 487): original description, figures of habitus, body parts, male genitalia.

#### Remarks.

Jeng (2008) reported a possible female of *M.kusakabei* from Myanmar.

### ﻿Genus Mimoochotyra

Taxon classificationAnimaliaColeopteraRhagophthalmidae

Pic, 1937

CB04E685-D7A4-5142-834F-477A85A3AA11

Mimoochotyra Pic, 1937: 137. Gender: feminine. Type species. Mimoochotyraocularis Pic, 1937; by monotypy.
Mimochotyra : McDermott 1964: 11 [unavailable name, incorrect subsequent spelling].
Mimotyra : Herring 1987: 158 [unavailable name, incorrect subsequent spelling not in prevailing usage].
Mimochotrya : Lawrence and Newton 1995: 857 [unavailable name, incorrect subsequent spelling not in prevailing usage].

#### Literature.

Pic (1937: 137): original description; McDermott (1964: 11, 51): revision [as *Mimochotyra* [sic!]]; McDermott (1966: 122): catalogue [as *Mimochotyra* [sic!]]; Mikšić and Mikšić (1966: 32): remark [as *Mimochotyra* [sic!]]; Herring (1987: 158): checklist [as *Mimotyra* [sic!]]; Lawrence and Newton (1995: 857): catalogue [as *Mimochotrya* [sic!]]; Kawashima (2000: 131): remark [as *Mimochotyra* [sic!]]; Bocakova et al. (2007: 484): molecular phylogeny [as *Mimochotyra* [sic!]]; Hunt et al. (2007: suppl.): molecular phylogeny [as *Mimochotyra* [sic!]]; Li et al. (2008a: 259): remark [as *Mimochotyra* [sic!]]; Li et al. (2008b: 495): review [as *Mimochotyra* [sic!]]; Li and Liang (2008: 111): remark [as *Mimochotyra* [sic!]]; Chen et al. (2010: 196): remark [as *Mimochotyra* [sic!]]; Costa and Zaragoza-Caballero (2010: 134): remark [as *Mimochotyra* [sic!]]; Kawashima et al. (2010: 135): book chapter [as *Mimochotyra* [sic!]]; Kundrata and Bocak (2011a: 59): remark [as *Mimochotyra* [sic!]]; Kundrata and Bocak (2011b: 370): molecular phylogeny [as *Mimochotyra* [sic!]]; Oba et al. (2011: 777): remark [as *Mimochotyra* [sic!]]; Kundrata et al. (2013: 202): molecular phylogeny; Kundrata et al. (2014: 167): molecular phylogeny; Bocak et al. (2016: suppl.): molecular phylogeny; Kundrata et al. (2016: 296): molecular phylogeny; Bocak et al. (2018: 4): molecular phylogeny; Kundrata et al. (2019: 1263): molecular phylogeny; Liu et al. (2020: 46): remark [as *Mimochotyra* [sic!]]. In addition to the aforementioned literature, this genus was included in a PhD thesis by Jeng (2008).

#### Remarks.

This genus currently contains a single described species from Java, Indonesia. According to Pic (1937), it is characterized by having serrate antennae with thickened median antennomeres, and relatively long elytra. The specimen identified as *Mimochotyra* [sic!] and used in the molecular phylogenetic analyses by Bocakova et al. (2007) and more recent studies needs serious re-examination, as it was collected in Malaysia.

### ﻿ Mimoochotyra ocularis

Taxon classificationAnimaliaColeopteraRhagophthalmidae

Pic, 1937

F72C2B9F-3A3E-5678-AA57-7F1E1E6F2956

Mimoochotyra ocularis Pic, 1937: 137.

#### Type depository.

Described based on an unknown number of specimens (probably only one). Syntype, male (NHMB).

#### Type locality.

Indonesia: Java, Gunung Raung [Raoeng], “Bajoekidoel” [detailed data taken from the syntype label; only “Java: Bajoekidoe” [sic!] in original description].

#### Distribution.

Indonesia (Java).

#### Literature.

Pic (1937: 137): original description; McDermott (1964: 51): revision [as *Mimochotyra* [sic!]]; McDermott (1966: 122): catalogue [as *Mimochotyra* [sic!]]; Li et al. (2008b: 496): review [as *Mimochotyra* [sic!]].

### ﻿Genus Monodrilus

Taxon classificationAnimaliaColeopteraRhagophthalmidae

Pic, 1921

1D2FB02D-5D5E-56A0-B6AB-0A3C3F91D8A5

Monodrilus Pic, 1921b: 12. Gender: masculine. Type species. Monodrilusmarginatus Pic, 1921; by monotypy.

#### Remarks.

*Monodrilus* has more or less serrate antennae and relatively long elytra, and currently contains two species from Indonesia (Java) and Vietnam, respectively, each in a monotypic subgenus. Following Wittmer (1944), we retain the concept of *Monodrilus* with two subgenera; however, Pic (1930b) already suggested *Dodecatomorpha* could be a separate genus. A proper taxonomic revision should be conducted to resolve the status of *Dodecatomorpha*.

### ﻿Subgenus Monodrilus

Taxon classificationAnimaliaColeopteraRhagophthalmidae

Pic, 1921

8F01BF07-D4C6-5884-9B1E-70C7EA9CBA07

Monodrilus Pic, 1921b: 12. Gender: masculine. Type species. Monodrilusmarginatus Pic, 1921; by monotypy.

#### Literature.

Pic (1921b: 12): original description; Pic (1928: 86): comparison with *Dodecatomorpha* Pic, 1928; Pic (1930b: 321): remark; Wittmer (1944: 212): catalogue; Lawrence et al. (2010b: 175): remark; Kundrata and Bocak (2011a: 57): remark; Janisova and Bocakova (2013: 3): remark. In addition to the aforementioned literature, *Monodrilus* was included in a PhD thesis by Jeng (2008).

#### Remarks.

This subgenus currently contains a single described species from Java, Indonesia.

### ﻿ Monodrilus marginatus

Taxon classificationAnimaliaColeopteraRhagophthalmidae

Pic, 1921

F26B13F4-5C8E-5512-859D-E3D3A411915F

Monodrilus marginatus Pic, 1921b: 12.

#### Type depository.

Described based on an unknown number of specimens. Syntype, male (MNHN).

#### Type locality.

Indonesia: Java.

#### Distribution.

Indonesia (Java).

#### Literature.

Pic (1921b: 12): original description; Pic (1928: 87): comparison with *Dodecatomorpharoberti* Pic, 1928; Wittmer (1944: 212): catalogue. In addition to the aforementioned literature, this species was included in a PhD thesis by Jeng (2008).

### ﻿Subgenus Dodecatomorpha

Taxon classificationAnimaliaColeopteraRhagophthalmidae

Pic, 1928

239836B9-2B1C-5852-852A-BA6FA2ACEA24

Dodecatomorpha Pic, 1928: 86 [as a subgenus of Monodrilus Pic, 1921]. Gender: feminine. Type species. Monodrilusroberti Pic, 1928 [in subgenus Dodecatomorpha]; by monotypy.

#### Literature.

Pic (1928: 86): original description; Pic (1930b: 321): remark; Wittmer (1944: 212): catalogue.

#### Remarks.

*Dodecatomorpha* currently contains a single described species from Vietnam.

### ﻿ Monodrilus (Dodecatomorpha) roberti

Taxon classificationAnimaliaColeopteraRhagophthalmidae

Pic, 1928

90CD2D3D-A7BE-5F3E-BB66-1075D0783AEF

Monodrilus roberti Pic, 1928: 86 [in subgenus Dodecatomorpha].

#### Type depository.

Described based on an unknown number of specimens. Three syntypes, males (MNHN).

#### Type locality.

Vietnam [“Darsa, en Cochinchine”].

#### Distribution.

Vietnam.

#### Literature.

Pic (1928: 86): original description; Pic (1930b: 321): remark; Wittmer (1944: 212): catalogue.

### ﻿Genus Pseudothilmanus

Taxon classificationAnimaliaColeopteraRhagophthalmidae

Pic, 1918

60995E64-A2AF-528A-BE13-BF96AB15E6B6

Pseudothilmanus Pic, 1918: 2. Gender: masculine. Type species: Pseudothilmanusalatus Pic, 1918; by monotypy.
Drilothilmanus Pic, 1918: 3. Type species: Drilothilmanusmarginatus, 1918; by monotypy. Synonymized by Kundrata and Bocak (2011a: 58).

#### Literature.

Pic (1918: 2, 3): original descriptions of *Pseudothilmanus* and *Drilothilmanus*, respectively; Wittmer (1944: 215): catalogue [also as *Drilothilmanus*]; Kundrata and Bocak (2011a: 58): revision, synonymization of *Drilothilmanus*; Liu et al. (2020: 46): remark; Roza (2020: 421): morphology, distribution, figures of male habitus, pronotum, and hind wing [2018 erroneously used as the date of the original description of this genus in figure caption]. In addition to the aforementioned literature, this genus was included in a PhD thesis by Roza (2022).

#### Remarks.

This genus has relatively long, serrate antennae and long elytra. It contains two species distributed in the Himalayas (India, Nepal).

### ﻿ Pseudothilmanus alatus

Taxon classificationAnimaliaColeopteraRhagophthalmidae

Pic, 1918

BF7F1F35-F3D7-5DDC-AC1B-6DAE0FEC59C0

Pseudothilmanus alatus Pic, 1918: 2.

#### Type depository.

Described based on an unknown number of specimens. Lectotype by present designation, with the following label data: “Type [red printed label] / Type [handwritten] / Nov. genus India [handwritten] / *Pseudothilmanusalatus* Pic [handwritten]” (treated as the holotype and figured by Kundrata and Bocak 2011a), male (MNHN).

#### Type locality.

India (without any further details).

#### Distribution.

India (Uttarakhand), Nepal.

#### Literature.

Pic (1918: 2): original description; Wittmer (1944: 215): catalogue; Kundrata and Bocak (2011a: 59): revision, figures of male habitus, antenna, pronotum, leg, elytral apex, pregenital segments, and genitalia; Roza (2020: 421): morphology, distribution, figures of male habitus, pronotum, and hind wing [2018 erroneously used as the date of the original description of this species in figure caption]. In addition to the aforementioned literature, this species was included in a PhD thesis by Roza (2022).

#### Remarks.

Roza (2020) listed Uttar Pradesh for the distribution of this species; however, it was based on the specimen from NHMUK mentioned by Kundrata and Bocak (2011a), which was collected in western Almora, Kumaon which lies in Uttarakhand (considered to be part of Uttar Pradesh prior to 2000).

### ﻿ Pseudothilmanus marginatus

Taxon classificationAnimaliaColeopteraRhagophthalmidae

Pic, 1918

998E819E-5DD3-5F78-904D-DF6AEB9C23A1

Drilothilmanus [as a subgenus of Pseudothilmanus] marginatus Pic, 1918: 3.
Pseudothilmanus marginatus : Kundrata and Bocak (2011a: 58).

#### Type depository.

Described based on an unknown number of specimens. Lectotype by present designation, with the following label data: “Type [red printed label] / Type [handwritten] / Darjeeling Juni Fruhstorfer leg. [printed] / *Drilothilmanusmarginatus* Pic [handwritten]” (treated as the holotype and figured by Kundrata and Bocak 2011a), male (MNHN).

#### Type locality.

India: West Bengal, Darjeeling.

#### Distribution.

India (West Bengal).

#### Literature.

Pic (1918: 3): original description; Wittmer (1944: 215): catalogue [as *Drilothilmanus*]; Kundrata and Bocak (2011a: 60): revision, figures of male habitus, antenna, pronotum, leg, and genitalia; Roza (2020: 422): morphology, distribution, figures of male habitus, pronotum, and hind wing [2018 erroneously used as the date of the original description of this species in figure caption].

### ﻿Genus Rhagophthalmus

Taxon classificationAnimaliaColeopteraRhagophthalmidae

Motschulsky, 1854

798AC722-75B9-542E-96DF-5B8ED0E4448F

Fig. 1E, F

Rhagophthalmus Motschulsky, 1854: 45. Gender: masculine. Type species: Rhagophthalmusscutellatus Motschulsky, 1854, by monotypy.
Ochotyra Pascoe, 1862: 323. Gender: feminine. Type species: Ochotyrasemiusta Pascoe, 1862: 323, by monotypy. Synonymized with Rhagophthalmus Motschulsky, 1854 by Wittmer in Wittmer and Ohba (1994: 342).
Ochotiza : Bourgeois 1903: 479 [unavailable name, incorrect subsequent spelling not in prevailing usage].
Ochrotyra : Lefroy 1909: 327 [unavailable name, incorrect subsequent spelling not in prevailing usage].
Rhagophthalma : Crowson 1981: 274 [unavailable name, incorrect subsequent spelling not in prevailing usage].
Ochotrya : Lawrence 1988: 15 [unavailable name, incorrect subsequent spelling not in prevailing usage].
Rhagophthalums : Suzuki 1997: 38 [unavailable name, incorrect subsequent spelling not in prevailing usage].
Ragophthalmus : Viviani et al. 1999: 8274 [unavailable name, incorrect subsequent spelling not in prevailing usage].
Rhagophtha : Chen 2003: 52 [unavailable name, incorrect subsequent spelling not in prevailing usage].
Rhagophtalmus : Stanger-Hall et al. 2007: 38 [unavailable name, incorrect subsequent spelling not in prevailing usage].
Rhagopthalmus : McKenna et al. 2015: 849 [unavailable name, incorrect subsequent spelling not in prevailing usage].

#### Literature.

Motschulsky (1854: 45): original description; Motschulsky (1859: 59): remark; Motschulsky (1861: 134): comparison with *Pachytarsus* Motschulsky, 1861; Pascoe (1862: 323): original description of *Ochotyra*; Gerstaecker (1863: 409): remark [as *Ochotyra*]; Gemminger (1869: 1647, 1655): catalogue [also as *Ochotyra*]; Marschall (1873: 223, 239): remark [also as *Ochotyra*]; Gorham (1881: 63): remark [as *Ochotyra*]; Olivier (1885: 372): species description; Heyden (1886: 286): remark; Fairmaire (1888: 25): species description [currently in *Menghuoius*]; Fairmaire (1889: 352): species description; Gorham (1890: 550): catalogue [as *Ochotyra*]; Bourgeois (1892: 236): distributional note; Cardon (1892: 238): checklist; Gorham (1895: 310): distributional note [also as *Ochotyra*]; Fairmaire (1896: 227): species descriptions [one currently in *Menghuoius*]; Fairmaire (1899: 624): species description; Olivier (1902: 87): catalogue; Bourgeois (1903: 479): distributional note [as *Ochotiza* [sic!]]; Gorham (1903: 330): distributional note [as *Ochotyra*]; Bourgeois (1905: 130): distributional record [as *Ochotyra*]; Olivier (1907: 63): catalogue [also as *Ochotyra*]; Olivier (1908: 17): remark; Lefroy (1909: 327): catalogue [also as *Ochrotyra* [sic!]]; Olivier (1910: 1): catalogue [also as *Ochotyra*]; Jakobson (1911: 687): catalogue; Olivier (1912: 467): revision, key [also as *Ochotyra*]; Pic (1916: 9): species description; Pic (1917: 3): species description; Lucas (1920: 567): catalogue; Pic (1921b: 18): species description [as *Ochotyra*]; Pic (1923: 25): catalogue; Handlirsch (1925: 589): catalogue; Pic (1925a: 17): species description; Pic (1925b: 72): species description; Winkler (1925: 522): catalogue; Pic (1937: 137): comparison of *Ochotyra* with *Mimoochotyra*; Wu (1937: 385): catalogue; Pic (1938: 15): checklist; Harvey (1952: 392): remark [also as *Ochotyra*]; Brues et al. (1954: 565): classification; Raj (1957: 788): larval biology, photograph of larvae; McDermott (1964: 11, 50): revision [also as *Ochotyra*]; McDermott (1966: 121): catalogue [also as *Ochotyra*]; Mikšić and Mikšić (1966: 32): remark [also as *Ochotyra*]; Nakane (1968: 3): remark; Crowson (1972: 52): remark; Herring (1978: 471): checklist; Lloyd (1978: 254): remark; Ohba (1980: 14): remark; Crowson (1981: 274): remark [as *Rhagophthalma* [sic!]]; Sivinski (1981: 168): bioluminescence; Herring (1987: 157): checklist [also as *Ochotyra*]; Lawrence (1988: 15): remark [also as *Ochotrya* [sic!]]; Wittmer and Ohba (1994: 341): review, synonymy of *Ochotyra* with *Rhagophthalmus*, figures of habitus and body parts; Lawrence and Newton (1995: 857): catalogue, remark [also as *Ochotrya* [sic!]]; Ohba (1995: 13): remark, bioluminescence; Branham (1996: 18): remark; Chen and Ho (1996: 46): distribution, figure of habitus; Ohba et al. (1996a: 1): morphology, biology; Ohba et al. (1996b: 30): remark; Nakane (1997: 36): remark; Ohba (1997a: 5): checklist; Ohba (1997b: 19): remark; Ohba (1997c: 51): breeding; Ohba et al. (1997: 25): remark; Suzuki (1997: 4): phylogeny, biology; Wittmer (1997: 257): species descriptions; Chen and Ho (1998: 34): bioluminescence; Kawashima (1998: 16): female morphology; Ohba (1998: 3): checklist, biology; Costa et al. (1999: 22): remark; Kawashima (1999: 141): remark; Viviani et al. (1999: 8274): remark [as *Ragophthalmus* [sic!]]; Goto and Kawashima (2000: 143): distributional remark; Jeng et al. (2000: 316): remark; Kawashima (2000: 131): taxonomy; Kim et al. (2000: 214): molecular phylogeny; Ohmiya et al. (2000: 32): luciferase; Viviani and Ohmiya (2000: 267): remark [as *Ragophthalmus* [sic!]]; Branham and Wenzel (2001: 565): phylogeny; Kawashima and Satô (2001: 423): species descriptions [also as *Ochotyra*]; Kobayashi et al. (2001: 1): embryogenesis; Viviani et al. (2001: 1287): bioluminescence [as *Ragophthalmus* [sic!]]; Hua (2002: 71): catalogue; Kawashima (2002: 492): remark; Kobayashi et al. (2002: 1): embryogenesis; O‘Keefe (2002: 182): remark; Ugarova and Brovko (2002: 322): bioluminescence; Viviani (2002: 1836): remark [as *Ragophthalmus* [sic!]]; Viviani et al. (2002: 538): remark [as *Ragophthalmus* [sic!]]; Branham and Wenzel (2003: 3): phylogeny, remark; Chen (2003: 52): morphology, bioluminescence [also as *Rhagophtha* [sic!]]; Hayashi and Suzuki (2003: 4): morphology, biology, phylogeny; Kawashima and Sugaya (2003: 353): species description, identification key; Kawashima et al. (2003: 255): catalogue [also *Ochotyra*]; Kobayashi et al. (2003: 19): embryogenesis, development; Satô and Kawashima (2003: 9): remark; DeCock (2004: 341): bioluminescence; Ohba (2004a: 226): bioluminescence, biology; Ohba (2004b: 6): bioluminescence, biology; Lau and Meyer-Rochow (2006: 20): morphology; Li et al. (2006: 818): molecular phylogeny; Arnoldi et al. (2007: 2): molecular phylogeny; Bocak (2007: 225): catalogue [also as *Ochotyra*]; Bocakova et al. (2007: 484): molecular phylogeny [as *Ochotyra*]; Geisthardt and Satô (2007: 234): catalogue [species *incertae sedis* in Lampyridae]; Hunt et al. (2007: suppl.): molecular phylogeny [as *Ochotyra*]; Lau et al. (2007: 27): eye morphology; Li et al. (2007: 197): mitochondrial genome, phylogeny; Sagegami-Oba et al. (2007: 105): molecular phylogeny; Stanger-Hall et al. (2007: 38): molecular phylogeny [also as *Rhagophtalmus* [sic!]]; Bocak et al. (2008: 2019): molecular phylogeny [as *Ochotyra*]; Dong et al. (2008: 479): phylogeny; Li and Liang (2008: 109): female morphology; Li et al. (2008a: 259): species descriptions, taxonomy, distribution [also as *Ochotyra*]; Li et al. (2008b: 494): review [also as *Ochotyra*]; Noguchi et al. (2008: 2): luciferase; Sheffield et al. (2008: 2500): mitochondrial genomes; Bogahawatta et al. (2009: 10): remark; Day et al. (2009: 93): remark; Levkanicova and Bocak (2009: 212): molecular phylogeny; Suzuki and Kobayashi (2009: 30): embryogenesis [also as *Ochotyra*]; Chen et al. (2010: 196): biology, bioluminescence; Costa and Zaragoza-Caballero (2010: 134): remark [also as *Ochotyra*]; Kawashima et al. (2010: 135): book chapter [also as *Ochotyra*]; Lawrence et al. (2010b: 173): remark; Bouchard et al. (2011: 326): family-group names catalogue; Kundrata and Bocak (2011a: 57): remark [also as *Ochotyra*]; Kundrata and Bocak (2011b: 370): molecular phylogeny [as *Ochotyra*]; Lawrence et al. (2011: 7): phylogeny; Oba et al. (2011: 777): biology, bioluminescence; Yiu (2011a: 14): remark; Yiu (2011b: 20): biology, bioluminescence; Amaral et al. (2012: 1262): luciferase, phylogeny; Ho et al. (2012: 1): species descriptions; Kazantsev (2012: 352): remark; Timmermans and Vogler (2012: 300): molecular phylogeny; Yiu (2012: 30): catalogue; Kundrata et al. (2013: 202): molecular phylogeny [as *Ochotyra*]; Yiu (2013: 101): biology, bioluminescence; Amaral et al. (2014: 415): molecular phylogeny [also as *Rhagophtalmus* [sic!]]; Hosoe et al. (2014: 331): chemical defence; Kundrata et al. (2014: 167): molecular phylogeny; Li et al. (2015: 269): catalogue; Martin et al. (2015: 519): molecular phylogeny; McKenna et al. (2015: 843): molecular phylogeny [also as *Rhagopthalmus* [sic!]]; Oba (2015: 99): bioluminescence; Amaral et al. (2016: 254): molecular phylogeny; Bocak et al. (2016: 3): molecular phylogeny; Kovalev and Kirejtshuk (2016: 205): remark; Kundrata et al. (2016: 296): molecular phylogeny; Wijekoon et al. (2016: 71): checklist [also as *Ochotyra*]; Amaral et al. (2017a: 674): mitogenome, phylogeny; Amaral et al. (2017b: 84): phylogeny; Amaral et al. (2017c: 157): phylogeny; Martin et al. (2017: 568): molecular phylogeny; Wang et al. (2017: 2): molecular phylogeny, transcriptome; Yiu (2017: 59): species description; Bocak et al. (2018: suppl): molecular phylogeny; Fallon et al. (2018: 8, 96): genomes, bioluminescence; Kusy et al. (2018a: 5): molecular phylogeny; Kusy et al. (2018b: 4): molecular phylogeny; Stanger-Hall et al. (2018: 8): remark; Yiu and Jeng (2018: 72): remark; Zhang et al. (2018: 3): molecular phylogeny; Amaral et al. (2019: 284): molecular phylogeny; Chen et al. (2019: 8): molecular phylogeny; He et al. (2019: 566): molecular phylogeny; Jeng (2019: 13): biofluorescence, biology; Kundrata et al. (2019: 1263): molecular phylogeny; Liu et al. (2019: 3183): mitogenomic phylogeny; Martin et al. (2019: 3): molecular phylogeny; Liu et al. (2020: 46): luciferase, phylogeny [also as *Ochotyra*]; Zhang et al. (2020: 5): molecular phylogeny, bioluminescence; Ge et al. (2021: 3): mitogenomic phylogeny; Lawrence et al. (2021: 456): wing morphology; Li et al. (2021b: 2): remark; Cai et al. (2022: 6): molecular phylogeny; Ge et al. (2022: 3): mitogenomic phylogeny; He et al. (2022: 4): mitogenomic phylogeny; Moreira et al. (2022: 7): luciferase, molecular phylogeny. In addition to the aforementioned literature, this genus was included in PhD theses by Ho (2002), Jeng (2008), and Roza (2022).

#### Remarks.

*Rhagophthalmus* is the most speciose genus in the family. It contains 34 species from South, East, and Southeast Asia. This genus is characterized by having deeply emarginate eyes and relatively short antennae (Fig. 1E, F). Wittmer in Wittmer and Ohba (1994) synonymized *Ochotyra* with *Rhagophthalmus*, and we follow this concept until a proper revision of the genus is carried out. On the other hand, *Menghuoius*, which was synonymized with *Rhagophthalmus* by Li et al. (2008a), is considered here a separate genus (see Remarks under *Menghuoius*).

### ﻿ Rhagophthalmus angulatus

Taxon classificationAnimaliaColeopteraRhagophthalmidae

Wittmer, 1997

60B37031-B17A-5538-A4B3-F08388D01BAB

Rhagophthalmus angulatus Wittmer, 1997: 258.

#### Type depository.

Holotype, male (NHMB). One paratype, male (NHMB).

#### Type locality.

China: East Hubei, 30 km NE Macheng, 500 m.

#### Distribution.

China (Hubei).

#### Literature.

Wittmer (1997: 258): original description, figures of male antenna and genitalia; Bocak (2007: 225): catalogue; Li et al. (2008a: 265): distribution; Li et al. (2008b: 496): review.

### ﻿ Rhagophthalmus beigansis

Taxon classificationAnimaliaColeopteraRhagophthalmidae

Ho in Ho et al. 2012

48719CAC-C1A6-5F9C-A0BA-4301F6563C52

Rhagophthalmus beigansis Ho in Ho et al. 2012: 4.

#### Type depository.

Holotype, male (TARI). Eight paratypes: four males, four females (ESRI, NMNS).

#### Type locality.

China/Taiwan, Lienchiang County, Beigan.

#### Distribution.

China/Taiwan.

#### Literature.

Ho et al. (2012: 4): original description, figures of male habitus, head, antenna and genitalia, and female habitus, head, and bioluminescence; Yiu (2017: 60): remark.

### ﻿ Rhagophthalmus brevipennis

Taxon classificationAnimaliaColeopteraRhagophthalmidae

Fairmaire, 1896

6CA53368-0FB8-5FBD-91A9-ACE2A1345C43

Rhagophthalmus brevipennis Fairmaire, 1896: 227.

#### Type depository.

Described based on an unknown number of specimens. Syntype, male (RMNH). Three additional male specimens (on one pin) from MNHN with labels different from the RMNH syntype are also labelled as “Type” but they probably represent a different species.

#### Type locality.

India: Maharashtra, Nagpur.

#### Distribution.

India (Maharashtra).

#### Literature.

Fairmaire (1896: 227): original description; Olivier (1902: 87): catalogue; Lefroy (1909: 327): catalogue; Olivier (1910: 1); catalogue; Olivier (1912: 470): revision; McDermott (1966: 121): catalogue; Li et al. (2008a: 265): distribution; Li et al. (2008b: 496): review.

### ﻿ Rhagophthalmus burmensis

Taxon classificationAnimaliaColeopteraRhagophthalmidae

Wittmer in Wittmer and Ohba 1994

3B4B5D4F-BA3F-5A25-8E70-09E711316B86

Rhagophthalmus burmensis Wittmer in Wittmer and Ohba 1994: 349.

#### Type depository.

Holotype, male (NHMB). Seven paratypes, males (NHMB).

#### Type locality.

Myanmar: Kambaiti.

#### Distribution.

Myanmar.

#### Literature.

Wittmer and Ohba (1994: 349): original description, drawings of male genitalia; Li et al. (2008a: 265): distribution; Li et al. (2008b: 496): review.

### ﻿ Rhagophthalmus confusus

Taxon classificationAnimaliaColeopteraRhagophthalmidae

Olivier, 1912

EE81D048-065A-5CC8-B54E-EFD34D5A9279

Rhagophthalmus confusus E. Olivier, 1912: 469, 471.
Rhagophthalmus confuses : Wijekoon et al. 2016: 71 [unavailable name, incorrect subsequent spelling not in prevailing usage].

#### Type depository.

Described based on an unknown number of specimens. One syntype, male (NHMUK). One probable syntype, male (MNHN).

#### Type locality.

Sri Lanka.

#### Distribution.

Sri Lanka.

#### Literature.

Olivier (1912: 469, 471): original description; Pic (1916: 9): comparison with *R.notaticollis*; McDermott (1966: 121): catalogue; Li et al. (2008a: 265): distribution; Li et al. (2008b: 496): review; Ho et al. (2012: 1): remark; Wijekoon et al. (2016: 71): checklist [as *R.confuses* [sic!]]. In addition to the aforementioned literature, this species was included in a PhD thesis by Roza (2022).

### ﻿ Rhagophthalmus elongatus

Taxon classificationAnimaliaColeopteraRhagophthalmidae

Wittmer in Wittmer and Ohba 1994

B6E42CE2-749C-5017-A5F4-C1A07C853EFB

Rhagophthalmus elongatus Wittmer in Wittmer and Ohba 1994: 348.

#### Type depository.

Holotype, male (NHMB).

#### Type locality.

China: Guangxi prov., Duyang Shan [“Mts. Toyen-chan”].

#### Distribution.

China (Guangxi).

#### Literature.

Wittmer and Ohba (1994: 348): original description, drawings of male genitalia; Kawashima and Satô (2001: 428, 430): comparison with *R.flavus* and *R.minutus*, respectively; Bocak (2007: 225): catalogue; Li et al. (2008a: 265): distribution; Li et al. (2008b: 496): review.

### ﻿ Rhagophthalmus filiformis

Taxon classificationAnimaliaColeopteraRhagophthalmidae

Olivier, 1912

64E102B6-489E-5616-BCBB-33A18EB52D95

Rhagophthalmus filiformis E. Olivier, 1912: 469, 470.

#### Type depository.

Described based on an unknown number of specimens. One syntype, male (NHMUK).

#### Type locality.

Sri Lanka.

#### Distribution.

Sri Lanka.

#### Literature.

Olivier (1912: 469, 470): original description; Pic (1925a: 17): comparison with *R.longipennis*; McDermott (1966: 121): catalogue; Kawashima and Satô (2001: 429): comparison with *R.minutus*; Li et al. (2008a: 265): distribution; Li et al. (2008b: 496): review; Ho et al. (2012: 1): remark; Wijekoon et al. (2016: 74): checklist.

### ﻿ Rhagophthalmus flavus

Taxon classificationAnimaliaColeopteraRhagophthalmidae

Kawashima & Satô, 2001

57D2C221-D994-58FA-8530-47DC3D5A0040

Rhagophthalmus flavus Kawashima & Satô, 2001: 424.

#### Type depository.

Holotype, male (NWU). One paratype, male (PCIK).

#### Type locality.

Myanmar: Dawna.

#### Distribution.

Myanmar, Thailand.

#### Literature.

Kawashima and Satô (2001: 424): original description, figures of male habitus, antenna, and genitalia; Li et al. (2008a: 265): distribution; Li et al. (2008b: 496): review; Ho et al. (2012: 9): comparison with *R.giallolateralus*.

### ﻿ Rhagophthalmus formosanus

Taxon classificationAnimaliaColeopteraRhagophthalmidae

Kawashima & Sugaya, 2003

E1DAF0C3-B8A9-545D-9734-CCC1DF400A63

Rhagophthalmus formosanus Kawashima & Sugaya, 2003: 354.

#### Type depository.

Holotype, male (NMNS). Two paratypes, males (PCIK).

#### Type locality.

China/Taiwan: Nantou Hsien, Meimu.

#### Distribution.

China/Taiwan.

#### Literature.

Kawashima and Sugaya (2003: 354): original description, identification key, figures of male habitus, antenna, and genitalia; Bocak (2007: 225): catalogue; Li et al. (2008a: 265): distribution; Li et al. (2008b: 496): review; Ho et al. (2012: 1): remark.

### ﻿ Rhagophthalmus fugongensis

Taxon classificationAnimaliaColeopteraRhagophthalmidae

Li & Liang in Li et al. 2008

F830C2A1-CE32-5E3C-A544-9BF41AF40AC0

Rhagophthalmus fugongensis Li & Liang in Li et al. 2008a: 260.

#### Type depository.

Holotype, male, No. 0058739 (KNHMZ). 22 paratypes: eight males and 13 females (KNHMZ), one paratype, male (YCM). Although Li et al. (2008a) stated in the original description that the “holotype and most paratypes are deposited in KIZ [now KNHMZ]; one paratype (male) is deposited in YCM” (Li et al. 2008a: 260), Li et al. (2015) listed only six paratypes (sex not mentioned) from KNHMZ, under the collection numbers 0058740–0058745.

#### Type locality.

China: Yunnan Province, Fugong County, Pihe, Wawa Village, 26.59398°N, 98.90819°E, 1263 m.

#### Distribution.

China (Yunnan).

#### Literature.

Li et al. (2008a: 260): original description; Li et al. (2008b: 496): review; Li et al. (2015: 269): catalogue.

### ﻿ Rhagophthalmus giallolateralus

Taxon classificationAnimaliaColeopteraRhagophthalmidae

Ho in Ho et al. 2012

739B19AD-4386-5174-B463-75C1BBC9433E

Rhagophthalmus giallolateralus Ho in Ho et al. 2012: 9.

#### Type depository.

Holotype, male (TARI). Four paratypes: two males and two females (ESRI, NMNS).

#### Type locality.

China/Taiwan, Lienchiang County, Dongjyu.

#### Distribution.

China/Taiwan.

#### Literature.

Ho et al. (2012: 9): original description, figures of male habitus, head, antenna, and genitalia, and female habitus, head, and luminous organ; Yiu (2017: 60): comparison with *R.motschulskyi*.

### ﻿ Rhagophthalmus gibbosulus

Taxon classificationAnimaliaColeopteraRhagophthalmidae

Fairmaire, 1899

3D8462F6-A51A-563A-AA08-73BFF4A423B3

Rhagophthalmus gibbosulus Fairmaire, 1899: 624.

#### Type depository.

Described based on an unknown number of specimens. No type specimen found in MNHN by RK.

#### Type locality.

Probably China, “Koua-Toun” (Fujian).

#### Distribution.

China (Fujian, ?Guangzhou, Shaanxi, Sichuan).

#### Literature.

Fairmaire (1899: 624): original description; Olivier (1902: 88): catalogue; Olivier (1910: 1): catalogue; Jakobson (1911: 687): catalogue; Olivier (1912: 470): revision; Winkler (1925: 522): catalogue; Wu (1937: 385): catalogue; McDermott (1966: 121): catalogue; Hua (2002: 71): catalogue; Bocak (2007: 225): catalogue; Li et al. (2008a: 263): distribution, description and figures of male genitalia; Li et al. (2008b: 496): review.

### ﻿ Rhagophthalmus hiemalis

Taxon classificationAnimaliaColeopteraRhagophthalmidae

Yiu, 2017

C9D29873-DD3F-5779-B4FE-36B996FB78DA

Rhagophthalmus hiemalis Yiu, 2017: 62.

#### Type depository.

Holotype, male (TLES). 15 paratypes: 10 males, five females (TLES).

#### Type locality.

China: Hong Kong, Tsuen Kam Au, 22.40728°N, 114.10357°E.

#### Distribution.

China (Hong Kong).

#### Literature.

Yiu (2017: 62): original description, figures of male and female habitus and male genitalia.

### ﻿ Rhagophthalmus jenniferae

Taxon classificationAnimaliaColeopteraRhagophthalmidae

Kawashima & Satô, 2001

E0787DC4-72C6-5E6B-A31E-490670EEC148

Rhagophthalmus jenniferae Kawashima & Satô, 2001: 430.
Rhagophthalmus jeniferae : Bocak 2007: 225 [unavailable name, incorrect subsequent spelling, not in prevailing usage].

#### Type depository.

Holotype, male (NWU). Three paratypes, males (NTU, PCIK).

#### Type locality.

China/Taiwan, Fenchihu, Chiai Hsien.

#### Distribution.

China/Taiwan.

#### Literature.

Kawashima and Satô (2001: 430): original description, figures of male habitus, antenna, and genitalia; Kawashima and Sugaya (2003: 353): remark, key; Bocak (2007: 225): catalogue [as *R.jeniferae* [sic!]]; Li et al. (2008a: 265): distribution; Li et al. (2008b: 496): review; Chen et al. (2010: 197): biology and bioluminescence, figures of male and female habitus, body parts, bioluminescence; Ho et al. (2012: 1): remark; Jeng (2019: 13): biofluorescence, biology, figures of larval and female habitus. In addition to the aforementioned literature, this species was mentioned in a PhD thesis by Ho (2002).

### ﻿ Rhagophthalmus kiangsuensis

Taxon classificationAnimaliaColeopteraRhagophthalmidae

Wittmer in Wittmer and Ohba 1994

75465716-710E-56F9-A31A-F95732394388

Rhagophthalmus kiangsuensis Wittmer in Wittmer and Ohba 1994: 346.
Rhagophthalmus kinagsuensis : Wittmer in Wittmer and Ohba 1994: 347 [unavailable name, incorrect original spelling (ICZN 1999, Art. 19.3); First Reviser (ICZN 1999, Art. 24.2): Bocak (2007: 225)].

#### Type depository.

Holotype, male (MNHN). One paratype, male (NHMB).

#### Type locality.

China: Jiangsu province (without further data).

#### Distribution.

China (Jiangsu).

#### Literature.

Wittmer and Ohba (1994: 346): original description, drawings of male genitalia; Bocak (2007: 225): catalogue; Li et al. (2008a: 265): distribution; Li et al. (2008b: 496): review.

### ﻿ Rhagophthalmus laosensis

Taxon classificationAnimaliaColeopteraRhagophthalmidae

Pic, 1917

B6A57FC6-DED1-50DC-971C-5CD1335BA355

Rhagophthalmus laosensis Pic, 1917: 3.

#### Type depository.

Described based on an unknown number of specimens. One syntype, male (MNHN).

#### Type locality.

Laos: Thakhek [“Taket”].

#### Distribution.

Laos.

#### Literature.

Pic (1917: 3): original description; Pic (1923: 29): catalogue; McDermott (1966: 122): catalogue; Li et al. (2008a: 265): distribution; Li et al. (2008b: 496): review.

### ﻿ Rhagophthalmus longipennis

Taxon classificationAnimaliaColeopteraRhagophthalmidae

Pic, 1925

7D0F9A94-F2F7-5642-B205-D6A9FB9F3F66

Rhagophthalmus longipennis Pic, 1925a: 17.
Ochotyra longipennis : Bocak 2007: 225.

#### Type depository.

Described based on an unknown number of specimens. Syntype, male (MNHN); syntype, male (NHMUK); four syntypes, males (NHMB).

Type localities. Only “Chine” in the original description (Pic 1925a). More detailed locality data available on the syntype labels: Sichuan, Kangding [“Tatsienlu”] (MNHN), Shaanxi, Qinling [“Kinling” or “Kinlung”] (NHMUK and NHMB, respectively).

#### Distribution.

China (Shaanxi, Sichuan).

#### Literature.

Pic (1925a: 17): original description; Wu (1937: 385): catalogue; McDermott (1966: 122): catalogue; Hua (2002: 71): catalogue; Bocak (2007: 225): catalogue [as *Ochotyralongipennis*]; Li et al. (2008a: 265): distribution; Li et al. (2008b: 496): review; Yiu (2017: 62): comparison with *R.hiemalis*.

#### Remarks.

Some previous authors erroneously considered 1923 as the year of original description of this species (as “Pic 1923: 29”); however, it was described as a new species in 1925 (Pic 1925a).

### ﻿ Rhagophthalmus lufengensis

Taxon classificationAnimaliaColeopteraRhagophthalmidae

Li & Ohba in Li et al. 2008

CD1D0C3C-D4BD-51CD-8413-A6BB35C2034C

Rhagophthalmus lufengensis Li, Ogoh, Ohba, Liang & Ohmiya, 2007: 196 [nomen nudum; published without description, unavailable name according to the ICZN (1999, Art. 13)].
Rhagophthalmus lufengensis Li & Ohba in Li et al. 2008a: 262.
Rhagophthalmus lufegensis : Amaral et al. 2014: 415 [unavailable name, incorrect subsequent spelling not in prevailing usage].

#### Type depository.

Holotype, male, No. 0058746 (KNHMZ). 11 paratypes: eight males and three females (KNHMZ, YCM). Li et al. (2015) listed three paratypes (two males, one female) from KNHMZ, under the collection numbers 0058747–0058749.

#### Type locality.

China: Yunnan Province, Lufeng County, Dajiuzhuang, 25.09774°N, 101.80204°E, 1827 m.

#### Distribution.

China (Yunnan).

#### Literature.

Li et al. (2007: 196): nomen nudum, mitochondrial genome, phylogeny; Arnoldi et al. (2007: 2): remark; Li et al. (2008a: 262): original description, figures of male and female habitus, male antenna, and male genitalia; Li et al. (2008b: 496): review; Sheffield et al. (2008: 2500): mitochondrial genome; Timmermans and Vogler (2012: 300): molecular phylogeny; Amaral et al. (2014: 415): molecular phylogeny [as *R.lufegensis* [sic!]]; Li et al. (2015: 269): catalogue; Amaral et al. (2016: 254): molecular phylogeny; Amaral et al. (2017a: 673): mitogenome, phylogeny [as *R.lufegensis* [sic!]]; Wang et al. (2017: 6): molecular phylogeny; Chen et al. (2019: 8): molecular phylogeny; He et al. (2019: 566): molecular phylogeny; Zhang et al. (2020: 5): molecular phylogeny; He et al. (2022: 4): mitogenomic phylogeny.

### ﻿ Rhagophthalmus minutus

Taxon classificationAnimaliaColeopteraRhagophthalmidae

Kawashima & Satô, 2001

16367371-C338-5095-BDF5-0622342B215D

Rhagophthalmus minutus Kawashima & Satô, 2001: 428.

#### Type depository.

Holotype, male (NWU). Three paratypes, males (two in NWU, one in PCIK).

#### Type locality.

Thailand: Kohn Kaen Province, “near Ban Lon, Lam Chee Yai”.

#### Distribution.

Thailand.

#### Literature.

Kawashima and Satô (2001: 428): original description, figures of male habitus, antenna, and genitalia; Kawashima and Sugaya (2003: 358): comparison with *R.formosanus*; Li et al. (2008a: 265): distribution; Li et al. (2008b: 496): review.

### ﻿ Rhagophthalmus motschulskyi

Taxon classificationAnimaliaColeopteraRhagophthalmidae

Olivier, 1912

3C041E30-B128-512F-ABAC-B12B5019F816

Rhagophthalmus motschulskyi E. Olivier, 1912: 469, 472.

#### Type depository.

Described based on an unknown number of specimens. Syntype, male (NHMUK).

#### Type locality.

China: Hong Kong.

#### Distribution.

China (Hong Kong).

#### Literature.

Olivier (1912: 469, 472): original description; Winkler (1925: 522): catalogue; McDermott (1966: 122): catalogue; Hua (2002: 71): catalogue; Bocak (2007: 225): catalogue; Li et al. (2008a: 265): distribution; Li et al. (2008b: 496): review; Yiu (2011a: 14): remark; Yiu (2011b: 20): biology and bioluminescence, figures of female bioluminescence; Ho et al. (2012: 1): remark; Yiu (2012: 30): catalogue, figures of habitus; Yiu (2013: 101): remark, bioluminescence; Yiu (2017: 60): redescription, figures of larva, pupa, and adults, and male genitalia.

### ﻿ Rhagophthalmus neoobscurus

Taxon classificationAnimaliaColeopteraRhagophthalmidae

Wittmer in Wittmer and Ohba 1994

3650E34A-00AD-5904-AF24-42C32B05E5F0

Ochotyra obscura Pic, 1921b: 18.
Rhagophthalmus neoobscurus Wittmer in Wittmer and Ohba 1994: 342 (replacement name for O.obscura Pic, 1921 (in Rhagophthalmus), not R.obscurus (Pic, 1917)).

#### Type depository.

Described based on an unknown number of specimens. One syntype, male (MNHN).

#### Type locality.

India (no further data). “Dekan India” written on the syntype label from MNHN.

#### Distribution.

India (no further data).

#### Literature.

Pic (1921b: 18): original description [as *Ochotyraobscura*]; McDermott (1966: 122): remark, catalogue [as *Ochotyraobscura*]; Wittmer and Ohba (1994: 342): taxonomy; Li et al. (2008a: 265): distribution; Li et al. (2008b: 496): review; Wijekoon et al. (2016: 74): checklist [as *Ochotyraobscura*].

#### Remarks.

Wijekoon et al. (2016) erroneously cited “P. Melong” instead of “Pic” as the author of *O.obscura*.

### ﻿ Rhagophthalmus notaticollis

Taxon classificationAnimaliaColeopteraRhagophthalmidae

Pic, 1916

3EC69DE1-B43E-5BC3-BA0E-2226726A0CDF

Rhagophthalmus notaticollis Pic, 1916: 9.
Rhagophthalmus notaticolis : Wijekoon et al. 2016: 74 [unavailable name, incorrect subsequent spelling not in prevailing usage].

#### Type depository.

Described based on an unknown number of specimens. One syntype, male (MNHN).

#### Type locality.

Sri Lanka.

#### Distribution.

Sri Lanka.

#### Literature.

Pic (1916: 9): original description; McDermott (1966: 122): catalogue; Li et al. (2008a: 265): distribution; Li et al. (2008b: 496): review; Ho et al. (2012: 1): remark; Wijekoon et al. (2016: 74): checklist [as *R.notaticolis* [sic!]].

#### Remarks.

Wijekoon et al. (2016) erroneously cited “P. Melong” instead of “Pic” as the author of *R.notaticollis*.

### ﻿ Rhagophthalmus obscurus

Taxon classificationAnimaliaColeopteraRhagophthalmidae

(Pic, 1917)

F1D5A4EC-167C-52AC-B73F-5FC4E7E50E0F

Rhagophthalmus tonkineus var. obscurus Pic, 1917: 4.
Rhagophthalmus tonkineus var. obscurus : Winkler 1925: 522 [unavailable name, incorrect subsequent spelling not in prevailing usage].
Rhagophthalmus tonkinensis var. obscurus : McDermott 1966: 122 [unavailable name, incorrect subsequent spelling not in prevailing usage].
Rhagophthalmus tokineus var. obscurus : Wittmer in Wittmer and Ohba 1994: 342 [unavailable name, incorrect subsequent spelling not in prevailing usage].
Rhagophthalmus obscurus : Wittmer in Wittmer and Ohba 1994: 342.

#### Type depository.

Described based on an unknown number of specimens. One syntype, male (MNHN).

#### Type locality.

Vietnam: Lào Cai [Tonkin: Lao Kay].

#### Distribution.

Vietnam.

#### Literature.

Pic (1917: 4): original description [as R.tonkineusvar.obscurus]; Pic (1923: 29): catalogue [as R.tonkineusvar.obscurus]; Winkler (1925: 522): catalogue [as R.tonkineusvar.obscursus [sic!]]; McDermott (1966: 122): catalogue [R.tonkinensisvar.obscurus [sic!]]; Wittmer and Ohba (1994: 342): taxonomy, drawings of male genitalia [also as R.tokineusvar.obscurus [sic!]]; Wittmer (1997: 258): remark; Li et al. (2008a: 265): distribution; Li et al. (2008b: 496): review.

### ﻿ Rhagophthalmus ohbai

Taxon classificationAnimaliaColeopteraRhagophthalmidae

Wittmer in Wittmer and Ohba 1994

F05EF9B5-CBEB-55E4-BF0D-E56D05B9C269

Rhagophthalmus ohbai Wittmer in Wittmer and Ohba 1994: 344.
Rhagophthalmus ohba : Branham and Wenzel 2001: 567 [unavailable name, incorrect subsequent spelling not in prevailing usage].

#### Type depository.

Holotype, male (YCM). Three paratypes, sex unknown (YCM), three paratypes, two males and one female (NHMB), two paratypes, sex unknown (NWU).

#### Type locality.

Japan: Okinawa Prefecture, Yaeyama Islands, Iriomote Island, Sonai.

#### Distribution.

Japan (Yaeyama Islands), Taiwan (Chen and Ho 1996, 1998; Ho et al. 2012).

#### Literature.

Wittmer and Ohba (1994: 344): original description, drawings of male and female habitus, male antenna and genitalia; Ohba (1995: 13): remark, bioluminescence; Chen and Ho (1996: 46): distribution, figure of habitus; Ohba et al. (1996a: 1): morphology, biology, figures of habitus, body details, and bioluminescence; Ohba et al. (1996b: 30): remark; Nakane (1997: 36): remark; Ohba (1997a: 5): checklist, biology, figures of larval, male and female habitus, male head, and female bioluminescence; Ohba (1997b: 19): remark; Ohba (1997c: 51): breeding, development, immature stages, figures of habitus; Ohba et al. (1997: 25): remark; Suzuki (1997: 4): phylogeny, biology; Wittmer (1997: 259): comparison with *R.angulatus*; Chen and Ho (1998: 34): bioluminescence; Kawashima (1998: 16): female morphology, drawing of female habitus; Ohba (1998: 3): checklist, biology; Costa et al. (1999: 22): remark; Viviani et al. (1999: 8274): remark [as *Ragophthalmus* [sic!]]; Goto and Kawashima (2000: 143): distribution; Kawashima (2000: 131): remarks; Kim et al. (2000: 214): molecular phylogeny; Ohmiya et al. (2000: 32): luciferase; Viviani and Ohmiya (2000: 267): remark [as *Ragophthalmus* [sic!]]; Branham and Wenzel (2001: 567): phylogeny [also as *R.ohba* [sic!]]; Kawashima and Satô (2001: 432): comparison with *R.jenniferae*; Kobayashi et al. (2001: 1): development, eggs; Viviani et al. (2001: 1287): bioluminescence [as *Ragophthalmus* [sic!]]; Kobayashi et al. (2002: 1): embryogenesis, figures of female habitus and bioluminescence; Ugarova and Brovko (2002: 322): bioluminescence; Viviani (2002: 1836): remark [as *Ragophthalmus* [sic!]]; Viviani et al. (2002: 538): remark [as *Ragophthalmus* [sic!]]; Branham and Wenzel (2003: 5): phylogeny [also as *R.ohba* [sic!]]; Chen (2003: 52): morphology, bioluminescence, figures of adult males and females, larva, and bioluminescence; Hayashi and Suzuki (2003: 4): morphology, biology, figure of mating; Kawashima and Sugaya (2003: 353): remark, identification key; Kawashima et al. (2003: 255): catalogue; Kobayashi et al. (2003: 19): embryogenesis, development; Satô and Kawashima (2003: 9): remark; DeCock (2004: 341): remark; Ohba (2004a: 226): bioluminescence, biology, figures of male and female habitus; Ohba (2004b: 6): bioluminescence, biology, figures of male and female habitus; Lau and Meyer-Rochow (2006: 20): eye morphology, figures of male and female head and eye; Li et al. (2006: 818): molecular phylogeny; Arnoldi et al. (2007: 2): molecular phylogeny; Bocak (2007: 225): catalogue; Geisthardt and Satô (2007: 234): catalogue [in Lampyridae*incertae sedis*]; Lau et al. (2007: 27): eye morphology of male; Li et al. (2007: 197): mitochondrial genome, phylogeny; Sagegami-Oba et al. (2007: 110): molecular phylogeny; Stanger-Hall et al. (2007: 38): molecular phylogeny [also as *Rhagophtalmus* [sic!]]; Li et al. (2008a: 259): comparison with *R.lufengensis* and *M.giganteus*, distribution; Li et al. (2008b: 496): review; Noguchi et al. (2008: 2): luciferase; Sheffield et al. (2008: 2500): mitochondrial genome; Suzuki and Kobayashi (2009: 30): embryogenesis, figure of egg; Chen et al. (2010: 203): habitus figure showing bioluminescence; Kawashima et al. (2010: 137): book chapter, figures of male and female habitus, and female ovipositor; Lawrence et al. (2011: 7): phylogeny, figure of female abdomen; Oba et al. (2011: 773): biology, bioluminescence, figures of male and female habitus, and female bioluminescence; Amaral et al. (2012: 1262): luciferase, phylogeny; Ho et al. (2012: 1): remarks, comparison with *R.beigansis*; Timmermans and Vogler (2012: 300): molecular phylogeny; Amaral et al. (2014: 415): molecular phylogeny; Hosoe et al. (2014: 331): chemical defence, figures of male and female habitus; Kundrata et al. (2014: 167): molecular phylogeny; Martin et al. (2015: 519): molecular phylogeny; Amaral et al. (2016: 254): molecular phylogeny; Bocak et al. (2016: suppl.): molecular phylogeny; Kundrata et al. (2016: 296): molecular phylogeny; Amaral et al. (2017a: 674): remark; Amaral et al. (2017b: 84): phylogeny; Amaral et al. (2017c: 157): phylogeny; Martin et al. (2017: 568): molecular phylogeny; Wang et al. (2017: 6): molecular phylogeny; Bocak et al. (2018: suppl.): molecular phylogeny; Fallon et al. (2018: 8): genomes, bioluminescence; Stanger-Hall et al. (2018: 8): remark; Chen et al. (2019: 8): molecular phylogeny; He et al. (2019: 566): molecular phylogeny; Kundrata et al. (2019: 1263): molecular phylogeny; Liu et al. (2019: 3183): mitogenomic phylogeny; Martin et al. (2019: 3): molecular phylogeny; Liu et al. (2020: 47): luciferase, phylogeny; Zhang et al. (2020: 5): molecular phylogeny, bioluminescence; Ge et al. (2021: 3): mitogenomic phylogeny; Ge et al. (2022: 3): mitogenomic phylogeny; He et al. (2022: 4): mitogenomic phylogeny; Moreira et al. (2022: 7): luciferase, molecular phylogeny. In addition to the aforementioned literature, this species was included in PhD theses by Ho (2002) and Jeng (2008).

### ﻿ Rhagophthalmus sausai

Taxon classificationAnimaliaColeopteraRhagophthalmidae

Wittmer, 1997

C4F415C2-EC04-5569-91FE-F82B3C55EBE0

Rhagophthalmus sausai Wittmer, 1997: 257.

#### Type depository.

Holotype, male (NHMB). Two paratypes, males (NHMB).

#### Type locality.

China: Guizhou, 60 km N Kaili, Shibing, Yuntai Shan.

#### Distribution.

China (Guizhou).

#### Literature.

Wittmer (1997: 257): original description, drawings of male antenna and genitalia; Bocak (2007: 225): catalogue; Li et al. (2008a: 265): distribution; Li et al. (2008b: 496): review.

### ﻿ Rhagophthalmus scutellatus

Taxon classificationAnimaliaColeopteraRhagophthalmidae

Motschulsky, 1854

BE3064F2-27B5-5EC4-B5C0-DE4690921853

Rhagophthalmus scutellatus Motschulsky, 1854: 45.

#### Type depository.

Holotype, male (ZMM).

#### Type locality.

China: Beijing.

#### Distribution.

China (Beijing, Fujian, Jiangsu/Shanghai).

#### Literature.

Motschulsky (1854: 45): original description; Motschulsky (1859: 59): remark, drawings of male habitus, lateral head, and leg; Gemminger (1869: 1655): catalogue; Olivier (1885: 372): comparison with *R.sumatrensis*; Heyden (1886: 286): remark; Fairmaire (1888: 25): comparison with *R.giganteus*; Fairmaire (1889: 353): comparison with *R.tonkineus*; Bourgeois (1892: 236): distributional note; Cardon (1892: 238): checklist; Fairmaire (1899: 624): comparison with *R.gibbosulus*; Olivier (1902: 88): catalogue; Olivier (1910: 1): catalogue; Jakobson (1911: 687): catalogue; Olivier (1912: 470): revision; Pic (1916: 9): comparison with *R.notaticollis*; Lucas (1920: 567): catalogue; Winkler (1925: 522): catalogue; Pic (1938: 15): checklist; McDermott (1964: 49): remark; McDermott (1966: 122): catalogue; Wittmer and Ohba (1994: 343): taxonomy, morphology, drawings of male genitalia; Wittmer (1997: 261): taxonomy, morphology, distribution, drawings of male genitalia; Kawashima and Satô (2001: 423): remark, comparison with *R.jenniferae*; Hua (2002: 71): catalogue; Kawashima et al. (2003: 255): remark, catalogue; Kawashima and Sugaya (2003: 358): remark, identification key; Bocak (2007: 225): catalogue; Li et al. (2008a: 264): comparison with *R.gibbosulus*, distribution; Li et al. (2008b: 494): review; Suzuki and Kobayashi (2009: 30): remark; Chen et al. (2010: 196): remark; Lawrence et al. (2011: 7): phylogeny; Yiu (2017: 60): remark, comparison with *R.hiemalis*; Lawrence et al. (2021: 456): wing morphology, figure of hind wing. In addition to the aforementioned literature, this species was included in a PhD thesis by Roza (2022).

### ﻿ Rhagophthalmus semisulcatus

Taxon classificationAnimaliaColeopteraRhagophthalmidae

Wittmer, 1997

701A1362-2971-5FAD-A325-598927A48F4A

Rhagophthalmus semisulcatus Wittmer, 1997: 259.

#### Type depository.

Holotype, male (NHMB). Five paratypes, males (NHMB). According to the original description (Wittmer 1997), there are only five paratypes; however, there are five additional specimens with different labels designated as paratypes in NHMB.

#### Type locality.

China: Yunnan: Yulong Shan, 27°10’N, 100°13’E, 3900 m.

#### Distribution.

China (Yunnan).

#### Literature.

Wittmer (1997: 259): original description, drawings of male antenna and genitalia; Bocak (2007: 225): catalogue; Li et al. (2008a: 264): distribution, biology, figure of female habitus; Li et al. (2008b: 496): review.

### ﻿ Rhagophthalmus semiustus

Taxon classificationAnimaliaColeopteraRhagophthalmidae

(Pascoe, 1862)

34B3ED28-B807-5E6D-A707-69560ADFB6AD

Ochotyra semiusta Pascoe, 1862: 323.
Rhagophthalmus (Ochrotyra [sic!]) semiusta [sic!]: Lefroy 1909: 327.
Rhagophthalmus semiustus : Wittmer in Wittmer and Ohba 1994: 342.

#### Type depository.

Holotype, male (NHMUK).

#### Type locality.

India: “Malabar”.

#### Distribution.

India (Karnataka, Kerala, Tamil Nadu) [“Malabar, Coromandel”], Sri Lanka.

#### Literature.

Pascoe (1862: 323): original description, drawing of male habitus [as *Ochotyra*]; Gerstaecker (1863: 409): remark [as *Ochotyra*]; Gemminger (1869: 1647): catalogue [as *Ochotyra*]; Gorham (1890: 550): catalogue; Gorham (1895: 310): distributional note, morphology [as *Ochotyra*]; Bourgeois (1903: 479): distributional note [as *Ochotiza* [sic!]]; Gorham (1903: 330): distributional note [as *Ochotyra*]; Bourgeois (1905: 130): distributional note [as *Ochotyra*]; Lefroy (1909: 327): catalogue [as *Rhagophthalmus* (*Ochrotyra* [sic!]) *semiusta* [sic!]]; Olivier (1910: 1): catalogue [as *Ochotyra*]; Pic (1921b: 18): comparison with *R.neoobscurus* [as *Ochotyra*]; McDermott (1964: 50): revision [as *Ochotyra*]; McDermott (1966: 122): catalogue [as *Ochotyra*]; Wittmer and Ohba (1994: 342): taxonomic remark [as *Ochotyra*]; Bocak (2007: 225): catalogue [as *Ochotyra*]; Li et al. (2008a: 265): distribution [as *R.semiusta* [sic!]]; Li et al. (2008b: 496): review [as *R.semiusta* [sic!]]; Wijekoon et al. (2016: 71): checklist [as *Ochotyra*]. In addition to the aforementioned literature, this species was included in a PhD thesis by Jeng (2008).

### ﻿ Rhagophthalmus sulcatus

Taxon classificationAnimaliaColeopteraRhagophthalmidae

Pic, 1925

34E64C06-789B-5251-8936-28D0C9C23DF5

Rhagophthalmus sulcatus Pic, 1925b: 72.

#### Type depository.

Described based on an unknown number of specimens. No type material was found in MNHN by RK.

#### Type locality.

India: West Bengal, Darjeeling.

#### Distribution.

India (West Bengal).

#### Literature.

Pic (1925b: 72): original description; McDermott (1966: 122): catalogue; Wittmer (1997: 261): comparison with other species; Bocak (2007: 225): catalogue; Li et al. (2008a: 265): distribution; Li et al. (2008b: 496): review.

#### Remarks.

This species could be a synonym of *R.sulcicollis* Olivier, 1912 (see Wittmer 1997 for more information).

### ﻿ Rhagophthalmus sulcicollis sulcicollis

Taxon classificationAnimaliaColeopteraRhagophthalmidae

Olivier, 1912

1DEE83C5-F591-5149-84A0-7E28506B61A7

Rhagophthalmus sulcicollis E. Olivier, 1912: 471.

#### Type depository.

Lectotype, male (NHMUK). Five paralectotypes, males (NHMUK) (although only four paralectotypes were reported by Wittmer 1997: 261). There are also two additional specimens in MNHN bearing the labels “lectotype” and “paralectotype”; however, they have locality label data that differ slightly from the original description.

#### Type locality.

China: Tibet/Xizang, Yalong, over 3000 m.

#### Distribution.

China (Tibet/Xizang).

#### Literature.

Olivier (1912: 471): original description; Winkler (1925: 522): catalogue; Pic (1925b: 72): comparison with *R.sulcatus*; McDermott (1966: 122): catalogue; Wittmer (1997: 261): taxonomy, morphology, drawings of male pronotum and genitalia; Hua (2002: 71): catalogue; Bocak (2007: 225): catalogue; Li et al. (2008a: 265): distribution; Li et al. (2008b: 496): review.

### ﻿ Rhagophthalmus sulcicollis bhutanensis

Taxon classificationAnimaliaColeopteraRhagophthalmidae

Wittmer, 1997

1599ADFD-D53F-5AB3-9D75-ECBEA9B53E0B

Rhagophthalmus sulcicollis subsp. bhutanensis Wittmer, 1997: 261.

#### Type depository.

Holotype, male (NHMB).

#### Type locality.

Bhutan: Karrumphe, 2700 m.

#### Distribution.

Bhutan.

#### Literature.

Wittmer (1997: 261): original description, drawings of male antenna and pronotum; Bocak (2007: 225): catalogue.

### ﻿ Rhagophthalmus sumatrensis

Taxon classificationAnimaliaColeopteraRhagophthalmidae

Olivier, 1885

810E5C7A-7E2A-5BB9-9AFD-DA592473158C

Rhagophthalmus sumatrensis Olivier, 1885: 372.

#### Type depository.

Described based on an unknown number of specimens. Three syntypes, males (MSNG).

#### Type locality.

Indonesia: Sumatra, Mt. Singalang.

#### Distribution.

Indonesia (Sumatra).

#### Literature.

Olivier (1885: 372): original description; Fairmaire (1889: 353): comparison with *R.tonkineus*; Olivier (1902: 88): catalogue; Olivier (1910: 1): catalogue; Olivier (1912: 470): revision, drawings of head, antenna, and tarsus; McDermott (1966: 122): catalogue; Wittmer (1997: 259): comparison with *R.angulatus*; Li et al. (2008a: 265): distribution; Li et al. (2008b: 496): review; Ho et al. (2012: 1): remark.

### ﻿ Rhagophthalmus tienmushanensis

Taxon classificationAnimaliaColeopteraRhagophthalmidae

Wittmer in Wittmer and Ohba 1994

1F8E5DC5-719B-5300-8795-8276BC0C8D33

Rhagophthalmus tienmushanensis Wittmer in Wittmer and Ohba 1994: 346.

#### Type depository.

Holotype, male (NHMB).

#### Type locality.

China: Zhejiang, Tianmushan.

#### Distribution.

China (Zhejiang, Shanghai).

#### Literature.

Wittmer and Ohba (1994: 346): original description, drawings of male genitalia; Bocak (2007: 225): catalogue; Li et al. (2008a: 265): distribution; Li et al. (2008b: 496): review.

### ﻿ Rhagophthalmus tonkineus

Taxon classificationAnimaliaColeopteraRhagophthalmidae

Fairmaire, 1889

B676B7AE-85C4-5DD8-A4FD-CD12B3CB5A45

Rhagophthalmus tonkineus Fairmaire, 1889: 352.
Rhagophthalmus tonkinensis : McDermott 1966: 122 [unavailable name, incorrect subsequent spelling not in prevailing usage].
Rhagophthalmus tokineus : Wittmer and Ohba 1994: 342 [unavailable name, incorrect subsequent spelling not in prevailing usage].

#### Type depository.

Described based on an unknown number of specimens. No type material was found in MNHN (Wittmer and Ohba 1994; RK, pers. obs.).

#### Type locality.

Vietnam [“Tonkin”].

#### Distribution.

Vietnam, China (Guangxi) (Li et al. 2008a); Laos (Pic 1923).

#### Literature.

Fairmaire (1889: 352): original description; Fairmaire (1896: 228): comparison with *R.brevipennis*; Olivier (1902: 88): catalogue; Olivier (1910: 1): catalogue; Olivier (1912: 470): revision; Pic (1917: 4): comparison with *R.obscurus*; Pic (1923: 29): catalogue, distributional note; Winkler (1925: 522): catalogue; McDermott (1966: 122): catalogue [as *R.tonkinensis* [sic!]]; Wittmer and Ohba (1994: 342): remark, taxonomy [as *R.tokineus* [sic!]]; Li et al. (2008a: 265): distribution [also as *R.tonkinensis* [sic!]]; Li et al. (2008b: 496): review [as *R.tonkinensis* [sic!]].

### ﻿ Rhagophthalmus xanthogonus

Taxon classificationAnimaliaColeopteraRhagophthalmidae

Olivier, 1912

272966E5-7C16-5F39-B200-67379585F633

Rhagophthalmus xanthogonus Olivier, 1912: 469, 471.
Rhagophthalmus xanthogenus : McDermott 1966: 122 [unavailable name, incorrect subsequent spelling not in prevailing usage].

#### Type depository.

Described based on an unknown number of male specimens. No type material was found in MNHN by RK.

#### Type locality.

China (no further data).

#### Distribution.

China (no further data).

#### Literature.

Olivier (1912: 469, 471): original description; Pic (1917: 4): comparison with *R.laosensis*; Winkler (1925: 522): catalogue; McDermott (1966: 122): catalogue [as *R.xanthogenus* [sic!]]; Hua (2002: 71): catalogue [as *R.xanthogenus* [sic!]]; Bocak (2007: 225): catalogue [as *R.xanthogenus* [sic!]]; Li et al. (2008a: 265): distribution [as *R.xanthogenus* [sic!]]; Li et al. (2008b: 496): review [as *R.xanthogenus* [sic!]].

##### ﻿Taxa removed from Rhagophthalmidae

### ﻿ Cydistus

Taxon classificationAnimaliaColeopteraRhagophthalmidae

Bourgeois, 1885 [Phengodidae: Cydistinae]

B63B8803-6966-5B25-A249-C4110F4B245C

Cydistus Bourgeois, 1885: 272. Type species. Cydistusreitteri Bourgeois, 1885; by monotypy.

#### Composition and distribution.

Six described species from Asia Minor, the Levant, Iraq, and Iran: *Cydistuschindaaricus* Bolívar y Pieltain, 1913, *C.escalerai* Bolívar y Pieltain, 1913, *C.nigripennis* Wittmer, 1979, *C.persicus* Bolívar y Pieltain, 1913, *C.reitteri* Bourgeois, 1885, and *C.zurcheri* Bourgeois, 1908 (Kundrata et al. 2019).

#### Remarks.

*Cydistus* was originally placed in Drilidae (Olivier 1910; Wittmer 1944). Later, Crowson (1955) hypothesized *Cydistus* might be an intermediate form between Karumiidae (currently a subfamily in Dascillidae) and Phengodidae. Although Paulus (1972) erected Cydistinae within Karumiidae for *Cydistus*, Crowson (1972) transferred this genus into the widely delimited Phengodidae, which also included Rhagophthalmidae. Lawrence and Newton (1995) and Bocak (2007) classified *Cydistus* in Phengodidae: Rhagophthalminae. Lawrence et al. (2010a, b) and Lawrence (2016) considered Cydistinae in Elateriformia*incertae sedis*. Finally, Kundrata et al. (2019) were the first to include Cydistinae in a molecular phylogenetic analysis, and found them sister to the New World Phengodidae, which are only distantly related to Rhagophthalmidae. This placement was confirmed by a morphology-based analysis by Roza (2022).

### ﻿ Luciola antipodum

Taxon classificationAnimaliaColeopteraRhagophthalmidae

Bourgeois, 1884 [Lampyridae: Luciolinae]

11B2DB26-6DFF-502A-9ACE-3A0E5129FF50

Luciola antipodum Bourgeois, 1884: 285.
Rhagophthalmus antipodum : Olivier 1902: 87; Fauvel, 1904: 140.
Bourgeoisia antipodum : Olivier 1908: 17.

#### Distribution.

New Caledonia, Solomon Islands.

#### Remarks.

This firefly species was originally described in *Luciola* Laporte, 1833 (Bourgeois 1884) and later transferred to *Rhagophthalmus* by Olivier (1902). The same author later placed this species in his new genus *Bourgeoisia* Olivier, 1908, and McDermott (1966) subsequently designated it the type species of this genus. *Bourgeoisia* is currently considered a synonym of *Luciola* (e.g., Ballantyne and Lambkin 2013; Ballantyne et al. 2019). For more information on *L.antipodum* see e.g., McDermott (1966); Ballantyne (1968); Ballantyne and Lambkin (2013); and Ballantyne et al. (2019).

### ﻿ Reductodrilus

Taxon classificationAnimaliaColeopteraRhagophthalmidae

Pic, 1943 [Lampyridae: Ototretinae]

7AACD42B-C4E0-5E18-A955-6F482FE6F6C5

Reductodrilus Pic, 1943: 9. Type species. Reductodrilusnigroapicalis Pic, 1943; by monotypy.

#### Composition and distribution.

Only a single species, *R.nigroapicalis* Pic, 1943 from northern Borneo (Malaysia: Sabah). Reductodrilusnigroapicalisvar.latetestaceus Pic, 1943 should have a subspecific status according to Article 45.6.4. of the Code (ICZN 1999).

#### Remarks.

*Reductodrilus* was initially placed in Drilidae (Pic 1943; Wittmer 1948). After most Drilidae genera were transferred to different families (e.g., Lampyridae, Lycidae, Omethidae, and Rhagophthalmidae) by Crowson (1972), *Reductodrilus* remained in an uncertain position. Kundrata and Bocak (2011a) listed it in Rhagophthalmidae in their revision of *Pseudothilmanus*. Probable syntypes of both subspecies of *R.nigroapicalis* are deposited in MNHN. Here, we tentatively transfer *Reductodrilus* to Lampyridae: Ototretinae based on its suboval and somewhat flattened body, antennae with 11 antennomeres which clearly extend beyond the posterior pronotal margin, head partially covered by pronotum, eyes clearly separated by frons, pronotum transverse, medially elevated, with anterior angles inconspicuous, rounded, and posterior angles projected posteriad (for more details see Janisova and Bocakova 2013). A detailed revision of this genus should improve our understanding of its systematic position.

## ﻿Discussion

Although Rhagophthalmidae have been known to entomologists for more than a century, their taxonomy and classification are still poorly known. The number of genera included in Rhagophthalmidae and also their placement within Elateroidea classification vary by source (e.g., McDermott 1966; Crowson 1972; Lawrence and Newton 1995; Kawashima et al. 2010; Kundrata and Bocak 2011a). In the last decade, Elateroidea systematic research has accelerated and the classification of the superfamily has experienced many taxonomic changes (e.g., Kundrata et al. 2014, 2019; Bocak et al. 2018; Kusy et al. 2018b, 2021), including the discoveries of two new recent families (Bocak et al. 2016; Rosa et al. 2020) and one new extinct family (Li et al. 2021b). However, only six new species of Rhagophthalmidae were described in three taxonomic papers in the same period (Ho et al. 2012; Kazantsev 2012; Yiu 2017). This is especially striking when compared to the most closely related family of Rhagophthalmidae (i.e., Phengodidae), where numerous taxonomic studies were published (e.g., Constantin 2014, 2016; Zaragoza-Caballero and Hernández 2014; Roza et al. 2017, 2018; Roza and Mermudes 2019, 2020; Vega-Badillo and Zaragoza-Caballero 2019; Vega-Badillo et al. 2020, 2021a, b), including not only descriptions of several new genera and species but also phylogenetic analyses of the group (Zaragoza-Caballero and Zurita-García 2015; Quintino 2017; Kundrata et al. 2019; Roza 2022). In Rhagophthalmidae, the most important research topics include taxonomic limits, phylogenetic relationships, accurate dating of the origin of the group, the evolution of bioluminescence and paedomorphosis, systematics of all genera (including revisions of already known species as well as descriptions of new taxa), descriptions of paedomorphic females and immature stages for all genera and species, and evaluating the distribution of the group at both generic and family levels.

### ﻿Phylogenetic relationships, origin, and monophyly of Rhagophthalmidae

The phylogenetic placement of Rhagophthalmidae within Elateroidea has been controversial based on morphology only (Crowson 1972; Lawrence 1988; Branham and Wenzel 2001; Lawrence et al. 2011), and Rhagophthalmidae were often placed either in or close to Lampyridae or Phengodidae. Molecular phylogenetic analyses using various datasets and analytical approaches repeatedly confirmed that Rhagophthalmidae are sister to Phengodidae (Bocakova et al. 2007; Kundrata and Bocak 2011b; Kundrata et al. 2014; Zhang et al. 2018; Douglas et al. 2021; Kusy et al. 2021; Cai et al. 2022). Both families share soft-bodied males with large eyes, often bipectinate antennae with 12 antennomeres, leathery elytra which are usually shortened and narrowed, larviform females, and larvae that possess bioluminescent organs and feed on millipedes (Kawashima et al. 2010; Zaragoza-Caballero and Hernández 2014; Kundrata et al. 2019). Kusy et al. (2021) defined the “lampyroid clade”, which contains Lampyridae, Phengodidae, Rhagophthalmidae, and Sinopyrophoridae. Fossil Cretophengodidae were probably also a part of that clade (Li et al. 2021b).

The date of the origin of Rhagophthalmidae is unclear, as there are no known fossils of the group. Generally, soft-bodied elateroids are rarely found as fossils, and to date, the most informative fossils are inclusions in various ambers. Cretophengodidae were described from mid-Cretaceous amber of northern Myanmar (ca. 99 Mya, Shi et al. 2012; Li et al. 2021b), and Kusy et al. (2021) reported unpublished Phengodidae from the same deposit. Kusy et al. (2021) summarized and reviewed the published molecular dating analyses of the elaterid-lampyroid clade, and showed that median estimates suggest the split of the Lampyridae, Phengodidae, and Rhagophthalmidae clade in the mid-Cretaceous. However, an earlier date is also possible (Kusy et al. 2021).

Another important issue is the monophyly of Rhagophthalmidae. The group was originally proposed only for *Dioptoma*, *Ochotyra*, and *Rhagophthalmus* (Olivier 1907, 1910), and later Pic (1937) added *Mimoochotyra*. This concept was adopted by McDermott (1964, 1966). Crowson (1972) transferred some Asian genera (*Cydistus*, *Diplocladon*, *Falsophrixothrix*) from Drilidae to Phengodidae, and these were later added to Rhagophthalmidae together with *Dodecatoma* (Lawrence and Newton 1995). *Cydistus* was later transferred to Phengodidae (Kundrata et al. 2019). The current concept of Rhagophthalmidae consists of males which have exactly 12 antennomeres, with antennomere III being longer than antennomere II, a telescopic abdomen that is usually narrowed apically, and females which are more or less larva-like. However, the monophyly of this group as currently defined has never been rigorously tested.

Several genera were included in molecular phylogenetic analyses, including *Bicladodrilus*, *Falsophrixothrix*, *Mimoochotyra*, and *Rhagophthalmus* (incl. *Ochotyra*) (Bocakova et al. 2007; Kundrata et al. 2014, 2019). These genera always formed a monophylum. However, it should be noted that at least the generic placements of specimens identified as *Bicladodrilus* sp. from China and *Mimoochotyra* sp. from Malaysia are dubious. As *Bicladodrilus*, *Bicladum*, and *Diplocladon* are similar in general appearance and possess biflabellate antennae, this generic complex is in need of revision. While there are no described *Bicladodrilus* species in China, a species of *Diplocladon* was recently described from Hong Kong (Yiu 2017). The single described species of *Mimoochotyra* is known from Java (Pic 1937; Wittmer 1944).

In his unpublished PhD thesis, Jeng (2008) focused on systematics and paedomorphosis (neoteny) in Lampyridae. He also included representatives of the rhagophthalmid genera *Dioptoma*, *Diplocladon*, *Dodecatoma*, *Falsophrixothrix*, *Menghuoius*, *Monodrilus*, and *Rhagophthalmus* (incl. *Ochotyra*) in his morphology-based analyses. These genera were monophyletic and sister to Phengodidae. In another unpublished PhD thesis, Roza (2022) focused on phylogenetic relationships of Phengodidae, and included *Bicladodrilus*, *Dioptoma*, *Diplocladon*, *Dodecatoma*, *Falsophrixothrix*, *Pseudothilmanus*, and *Rhagophthalmus* in his morphology-based analyses. These genera formed a monophylum in all analyses performed. A phylogenomic analysis including representatives (ideally type species) of all rhagophthalmid genera would be a valuable assessment of the monophyly of the group.

### ﻿Bioluminescence and paedomorphosis in Rhagophthalmidae

Within Coleoptera, bioluminescence can be found almost exclusively within the so-called “elaterid-lampyroid clade”, including Elateridae, Lampyridae, Phengodidae, Rhagophthalmidae, and Sinopyrophoridae, and probably the extinct Cretophengodidae (Oba et al. 2011; Fallon et al. 2018; Bi et al. 2019; Li et al. 2021b; Kusy et al. 2021; Powell et al. 2022). In Phengodidae, all known larvae and females are bioluminescent, as are males of some species (Costa and Zaragoza-Caballero 2010). Bioluminescence is hypothesized for lineages in which larvae and females are unknown (e.g., Cydistinae; Kundrata et al. 2019). All known larvae and females of Rhagophthalmidae are bioluminescent. Both larvae and females were reported for *Diplocladon* (in fact, it is probably *Haplocladon*; see Remarks under both genera), *Menghuoius*, and *Rhagophthalmus*, whereas only females are known for *Dioptoma* (Green 1913; Gahan 1925; Coblentz and Hughes 1926; Ridley 1934; Haneda 1950; Raj 1957; Lloyd 1971, 1978; Wittmer and Ohba 1994; Ohba et al. 1996a; Kawashima 2000; Jeng 2008; Li and Liang 2008; Li et al. 2008a; Kawashima et al. 2010). Males of at least some genera (e.g., *Dioptoma* and *Rhagophthalmus*) also emit light (Kawashima et al. 2010). At least in some cases, however, there are doubts about the correct genus identification of larvae or females. For example, Jeng (2008) suggested that the giant larviform female from Yunnan, China, identified as *Diplocladon* by Li and Liang (2008) is “likely of *Menghuoiusgiganteus* or the other related species described from there” (Jeng 2008: 135). The correct identification of larviform females is, however, very important for conclusions on the evolution of morphological modifications caused by paedomorphosis (e.g., Jeng 2008; Kawashima et al. 2010). This should be possible by e.g., rearing both sexes of the same species from larvae, finding a mating couple, or by the use of DNA barcoding. Information on the life-history and biology of most genera of Rhagophthalmidae is minimal or entirely absent. Further research should be conducted to confirm the presence of bioluminescence in the remaining rhagophthalmid genera.

Elateroid beetles are well-known not only for bioluminescence but also for morphological modifications caused by paedomorphosis (Crowson 1972; Cicero 1988; Bocak et al. 2008; Ferreira and Ivie 2022). In Elateroidea, mainly adult females are more or less modified, with a gradual series of morphological modifications across many families (Elateridae, Jurasaidae, Lampyridae, Lycidae, etc.), ranging from taxa that possess only a slightly softer body cuticle and a more relaxed abdomen through a number of intermediate stages, with variously reduced mouthparts, antennae, elytra, hind wings, and/or parts of the thorax, and a higher number of free abdominal ventrites, to taxa which are highly larviform (Bocakova et al. 2007; Bocak et al. 2008; Ferreira et al. 2019, 2020, 2022; Kundrata and Bocak 2019; Rosa et al. 2020; Ferreira and Ivie 2022). In both Phengodidae and Rhagophthalmidae, all known females are highly paedomorphic, being wingless and larva-like (Costa and Zaragoza-Caballero 2010; Kawashima et al. 2010). In Rhagophthalmidae, females are known only for *Dioptoma*, *Diplocladon* (*Haplocladon*?), *Menghuoius*, and *Rhagophthalmus* (Haneda 1950; Harvey 1952; Ohba 1997c; Kawashima 1998; Chen 2003; Jeng 2008; Li and Liang 2008; Li et al. 2008a); however, only those of *Diplocladon* (*Haplocladon*?) and *Rhagophthalmus* are described in detail (Kawashima et al. 2010). Interestingly, females of both genera exhibit different degrees of paedomorphic modifications, with *Diplocladon* (*Haplocladon*?) being completely larviform (including having stemmata, antennae with three antennomeres, tibiotarsus with a single pretarsal claw, and missing ovipositor) and *Rhagophthalmus* being incompletely larviform (having compound eyes, antennae with six or seven antennomeres, tarsi with five tarsomeres and two pretarsal claws, and ovipositor; Kawashima et al. 2010). Similar cases of different levels of morphological modifications in females of different genera were also reported for e.g., Elateridae (*Drilus* Olivier, 1790 being more paedomorphic than *Omalisus* Geoffroy, 1762 or *Cebrio* Olivier, 1790; Kundrata and Bocak 2019), Jurasaidae (*Jurasai*Rosa et al., 2020 being more paedomorphic than *Tujamita*Rosa et al., 2020; Rosa et al. 2020), and Lampyridae (*Lamprigera* Motschulsky, 1853 or *Stenocladius* Fairmaire, 1878 being more paedomorphic than *Lampyris* Geoffroy, 1762 or *Lamprohiza* Motschulsky, 1853; Ohba et al. 1997; Dong et al. 2021). It would be, therefore, very interesting to discover and describe in detail the females of all remaining rhagophthalmid genera.

### ﻿Generic classification and systematics of Rhagophthalmidae

It is clear from the above text that the classification and systematics of Rhagophthalmidae is in a very poor state of knowledge. Species of *Bicladodrilus*, *Bicladum*, *Falsophrixothrix*, *Mimoochotyra*, and *Monodrilus* have not been taxonomically treated since their descriptions, and their names have usually appeared only in catalogues, if at all. Taxonomic revisions are urgently needed for all genera currently included in Rhagophthalmidae with the exception of *Pseudothilmanus*, which was revised recently (Kundrata and Bocak 2011a). Although the most species-rich genus *Rhagophthalmus* received some taxonomic attention in the last decades (e.g., Wittmer and Ohba 1994; Wittmer 1997; Kawashima and Satô 2001; Kawashima and Sugaya 2003; Li et al. 2008a; Ho et al. 2012; Yiu 2017), a comprehensive revision is still needed.

Due to a scarcity of information on the morphology of most rhagophthalmid taxa, an identification key which would help taxonomists to recognize genera and species in collections and subsequently enhance knowledge on their diversity, variability, and distributions, is also missing. Most importantly, it is necessary to delimit generic boundaries in some problematic generic complexes. For example, *Bicladodrilus*, *Bicladum*, and *Diplocladon* share biflabellate antennae and relatively long elytra, and are not clearly distinguished from one other. Detailed taxonomic studies should also be conducted to revise the status of *Ochotyra* (currently a synonym of *Rhagophthalmus*) and *Menghuoius* (currently a separate genus but treated by some authors as a synonym of *Rhagophthalmus*). Some genera contain species which are probably not congeneric with their type species (e.g., some *Dodecatoma* spp. resemble *Pseudothilmanus* more than *D.bicolor*), and e.g., *Dodecatomatestaceiceps* should be removed from Rhagophthalmidae after a detailed revision. Taxonomic attention should be given not only to currently described taxa, but also to numerous undescribed Rhagophthalmidae mainly from Southeast Asia, which are housed in various institutional and personal collections (RK pers. obs.).

Taxonomic revisions are usually hampered by missing, lost, or otherwise unavailable type specimens, especially in long-neglected groups, such as Rhagophthalmidae. However, the vast majority of name-bearing rhagophthalmid type specimens are available in European and Asian museum collections. To date, we have been unable to locate name-bearing type specimens of only five species described by either Pic, Fairmaire, or Olivier, four of which belong to *Rhagophthalmus*. Name-bearing type specimens of species in 10 smaller genera are each deposited in one to three museum collections only; however, those of *Dodecatoma* and *Rhagophthalmus* are in seven and 12 institutions, respectively.

### ﻿Distribution of Rhagophthalmidae

Rhagophthalmidae are distributed in the Oriental realm and the Palaearctic bioregion of East Asia, in the area from Afghanistan and Pakistan, through the Himalayas, Indian Peninsula, Sri Lanka, China, and mainland Southeast Asia, to Sumatra, Java, Bali, Borneo, and the Philippines. The center of genus-level diversity of Rhagophthalmidae lies in Southeast Asia. Nine out of 12 genera have at least one species distributed in Southeast Asia, with five genera (i.e., *Bicladodrilus*, *Bicladum*, *Falsophrixothrix*, *Mimoochotyra*, and *Monodrilus*) being endemic to the region. However, this only accounts for approximately one third of described species. The genera *Dodecatoma* and *Pseudothilmanus* are known only from the Himalayas and surrounding regions (one species and one subspecies of *Dodecatoma* from Southeast Asia should be removed from that genus), and *Dioptoma* is endemic to the Indian Peninsula and Sri Lanka. Regarding the most species-rich genus *Rhagophthalmus*, only seven out of 34 species are known from Southeast Asia, including only a single species from the Greater Sunda Islands. Another seven species are known from the Indian Peninsula and Sri Lanka, and the remaining majority of species are distributed in mainland China and among the islands of East Asia.

Interestingly, in the eastern part of their distribution, Rhagophthalmidae have remained within the boundaries of the Sunda Shelf and the Philippines, i.e, west of the originally proposed Wallace Line, which was demarcated to separate Indo-Malayan (Oriental) and Austro-Malayan (Australasian) realms (Wallace 1863; Voris 2000; Lohman et al. 2011). The Sunda Shelf is a southward expansion of the continental shelf of Southeast Asia that was intermittently exposed by lowered sea levels during the Pleistocene (Hall 1998; Voris 2000; Lomolino et al. 2017). The Wallace line separates Bali and Borneo on the west from Lombok and Sulawesi on the east. It is a strong dispersal barrier to many terrestrial animals because of deep oceanic trenches (Lomolino et al. 2017). Rhagophthalmidae have a limited dispersal propensity due to their highly modified larviform females and, therefore, it is not surprising that they remained within the boundaries of the continental shelf of Southeast Asia, with a single described species from the Philippines. Additionally, it should be noted that there are no Rhagophthalmidae from east of the Wallace Line among the extensive material of non-type specimens (including numerous new species) that reside in major European museums and several personal collections, which the first author examined for a planned generic revision of the group. A single, unreported rhagophthalmid species from Bali is the closest that the family has been observed to the Wallace Line. A taxonomic revision of Rhagophthalmidae will further improve our knowledge of the distributions of individual genera and their species, some of which are currently known only from a single specimen.

## ﻿Conclusions

Here we provide the first comprehensive catalogue of the currently defined Rhagophthalmidae. The only catalogues of the group were those of Olivier (1910) and McDermott (1966; Rhagophthalminae as a subfamily of Lampyridae) but they contained only three genera and nine species, and four genera and 21 species, respectively. Here, we recognize 12 genera and 66 species. However, all genera but *Pseudothilmanus* urgently need taxonomic revisions, and numerous new species await formal descriptions. The phylogenetic position of Rhagophthalmidae as a sister group to Phengodidae is now generally accepted; however, interrelationships within the group and generic classification remain poorly known. Although morphology-based analyses in two PhD studies that were focused on related families (Jeng 2008; Roza 2022) confirm the monophyly of currently circumscribed Rhagophthalmidae, molecular analysis including representatives of all genera would be desirable. Additionally, little information is known of the biology of the group. Although various studies have been published on the ontogeny, biology, and behaviour of the most common genus *Rhagophthalmus*, there is virtually nothing known about the majority of other genera. Because known females in Rhagophthalmidae are highly morphologically modified and remain larviform as adults, they are interesting subjects for researching the evolution of paedomorphosis in Elateroidea. However, only a few have been studied in detail (Kawashima et al. 2010). Additionally, all known larvae and females (and some males) are bioluminescent, and therefore are an important source of information for research on the evolution of bioluminescence in beetles. However, this phenomenon is also understudied in Rhagophthalmidae, as larvae and females are unknown for most genera. Discoveries, field observations, morphological studies, and correct genus and species identifications of larvae and larviform females of Rhagophthalmidae are therefore crucial not only for our improved knowledge of the diversity, systematics, and morphology of the group, but also for a better understanding of the evolution of paedomorphosis and bioluminescence in Elateroidea and beetles in general.

## Supplementary Material

XML Treatment for Rhagophthalmidae

XML Treatment for Bicladodrilus

XML Treatment for Bicladodrilus bakeri

XML Treatment for Bicladodrilus laticollis

XML Treatment for Bicladum

XML Treatment for Bicladum mjobergi

XML Treatment for Bicladum multipunctatum

XML Treatment for Dioptoma

XML Treatment for Dioptoma adamsii

XML Treatment for Dioptoma atripennis

XML Treatment for Diplocladon

XML Treatment for Diplocladon atripenne

XML Treatment for Diplocladon hasseltii hasseltii

XML Treatment for Diplocladon hasseltii testaceum

XML Treatment for Dodecatoma

XML Treatment for Dodecatoma bicolor

XML Treatment for Dodecatoma fuscicornis fuscicornis

XML Treatment for Dodecatoma fuscicornis testaceicornis

XML Treatment for Dodecatoma gracilis

XML Treatment for Dodecatoma parvicornis

XML Treatment for Dodecatoma riedeli

XML Treatment for Dodecatoma saluki

XML Treatment for Dodecatoma schmidti

XML Treatment for Dodecatoma testaceiceps

XML Treatment for Falsophrixothrix

XML Treatment for Falsophrixothrix costata

XML Treatment for Falsophrixothrix flava

XML Treatment for Falsophrixothrix humeralis

XML Treatment for Falsophrixothrix javana

XML Treatment for Falsophrixothrix punctata

XML Treatment for Falsophrixothrix pygmaea

XML Treatment for Haplocladon

XML Treatment for Haplocladon gorhami

XML Treatment for Haplocladon indicum

XML Treatment for Menghuoius

XML Treatment for Menghuoius giganteus

XML Treatment for Menghuoius ingens

XML Treatment for Menghuoius kusakabei

XML Treatment for Mimoochotyra

XML Treatment for Mimoochotyra ocularis

XML Treatment for Monodrilus

XML Treatment for Monodrilus

XML Treatment for Monodrilus marginatus

XML Treatment for Dodecatomorpha

XML Treatment for Monodrilus (Dodecatomorpha) roberti

XML Treatment for Pseudothilmanus

XML Treatment for Pseudothilmanus alatus

XML Treatment for Pseudothilmanus marginatus

XML Treatment for Rhagophthalmus

XML Treatment for Rhagophthalmus angulatus

XML Treatment for Rhagophthalmus beigansis

XML Treatment for Rhagophthalmus brevipennis

XML Treatment for Rhagophthalmus burmensis

XML Treatment for Rhagophthalmus confusus

XML Treatment for Rhagophthalmus elongatus

XML Treatment for Rhagophthalmus filiformis

XML Treatment for Rhagophthalmus flavus

XML Treatment for Rhagophthalmus formosanus

XML Treatment for Rhagophthalmus fugongensis

XML Treatment for Rhagophthalmus giallolateralus

XML Treatment for Rhagophthalmus gibbosulus

XML Treatment for Rhagophthalmus hiemalis

XML Treatment for Rhagophthalmus jenniferae

XML Treatment for Rhagophthalmus kiangsuensis

XML Treatment for Rhagophthalmus laosensis

XML Treatment for Rhagophthalmus longipennis

XML Treatment for Rhagophthalmus lufengensis

XML Treatment for Rhagophthalmus minutus

XML Treatment for Rhagophthalmus motschulskyi

XML Treatment for Rhagophthalmus neoobscurus

XML Treatment for Rhagophthalmus notaticollis

XML Treatment for Rhagophthalmus obscurus

XML Treatment for Rhagophthalmus ohbai

XML Treatment for Rhagophthalmus sausai

XML Treatment for Rhagophthalmus scutellatus

XML Treatment for Rhagophthalmus semisulcatus

XML Treatment for Rhagophthalmus semiustus

XML Treatment for Rhagophthalmus sulcatus

XML Treatment for Rhagophthalmus sulcicollis sulcicollis

XML Treatment for Rhagophthalmus sulcicollis bhutanensis

XML Treatment for Rhagophthalmus sumatrensis

XML Treatment for Rhagophthalmus tienmushanensis

XML Treatment for Rhagophthalmus tonkineus

XML Treatment for Rhagophthalmus xanthogonus

XML Treatment for Cydistus

XML Treatment for Luciola antipodum

XML Treatment for Reductodrilus
